# Gut microbiota and metabolic profiles in adults with unclassified diabetes: a cross-sectional study

**DOI:** 10.3389/fendo.2024.1440984

**Published:** 2024-11-11

**Authors:** Juan Zhang, Lei Wu, Zhongyun Zhang, Danjie Li, Rulai Han, Lei Ye, Yifei Zhang, Jie Hong, Weiqiong Gu

**Affiliations:** ^1^ Department of Endocrine and Metabolic Diseases, Shanghai Institute of Endocrine and Metabolic Diseases, Ruijin Hospital, Shanghai Jiao Tong University School of Medicine, Shanghai, China; ^2^ Shanghai National Clinical Research Center for Metabolic Diseases, Key Laboratory for Endocrine and Metabolic Diseases of the National Health Commission of the PR China, Shanghai National Center for Translational Medicine, Shanghai, China

**Keywords:** diabetes mellitus, gut microbiome, metagenome, metabolites analysis, correlation

## Abstract

**Aims:**

Our study, employing a multi-omics approach, aimed to delineate the distinct gut microbiota and metabolic characteristics in individuals under 30 with unclassified diabetes, thus shedding light on the underlying pathophysiological mechanisms

**Methods:**

This age- and sex-matched case-control study involved 18 patients with unclassified diabetes, 18 patients with classic type 1 diabetes, 13 patients with type 2 diabetes, and 18 healthy individuals. Metagenomics facilitated the profiling of the gut microbiota, while untargeted liquid chromatography-mass spectrometry was used to quantify the serum lipids and metabolites.

**Results:**

Our findings revealed a unique gut microbiota composition in unclassified diabetes patients, marked by a depletion of *Butyrivibrio proteoclasticus* and *Clostridium* and an increase in *Ruminococcus torques* and *Lachnospiraceae bacterium 8_1_57FAA*. Comparative analysis identified the combined marker panel of five bacterial species, seven serum biomarkers, and three clinical parameters could differentiate patients with UDM from HCs with an AUC of 0.94 (95% CI 0.85–1). Notably, the gut microbiota structure of patients with unclassified diabetes resembled that of type 2 diabetes patients, especially regarding disrupted lipid and branched-chain amino acid metabolism.

**Conclusions:**

Despite sharing certain metabolic features with type 2 diabetes, unclassified diabetes presents unique features. The distinct microbiota and metabolites in unclassified diabetes patients suggest a significant role in modulating glucose, lipid, and amino acid metabolism, potentially influencing disease progression. Further longitudinal studies are essential to explore therapeutic strategies targeting the gut microbiota and metabolites to modify the disease trajectory.

## 1 Introduction

The global incidence of diabetes mellitus has risen dramatically, signifying a major public health dilemma in the twenty-first century (1). Diabetes is characterized by increasing heterogeneity, resulting in a broad spectrum of clinical manifestations and a more diverse range of diabetic subgroups. The increasing incidence of overweight and obesity in individuals with type 1 diabetes mellitus (T1DM) (2), coupled with the occurrence of ketosis or ketoacidosis in types beyond T1DM, further complicates the classification process, especially at the initial diagnosis stage. Consequently, the classification and management of diabetes will become increasingly challenging (3). To underscore this complexity, the World Health Organization (WHO) introduced the category of unclassified diabetes (UDM) in 2019 (4). Nevertheless, the distinctive characteristics and etiological factors of UDM have not been fully elucidated.

Emerging evidence indicates a distinct imbalance in the gut microbiota of patients with childhood-onset T1DM and type 2 diabetes mellitus (T2DM) (5, 6). In childhood-onset T1DM, a reduced *Firmicutes*-to-*Bacteroides* ratio is common, as is an increased prevalence of *Bacteroides* and *Blautia* (7, 8). Research in T1DM animal models suggests that the gut microbiota may influence the autoimmune destruction of pancreatic beta cells by modulating toll-like receptor 2/4 signaling, Th17 cells in the intestinal mucosa, sex hormone levels, and the secretion of pancreatic antibacterial peptides (9–11). Conversely, T2DM patients often exhibit a decrease in butyrate-producing bacteria, notably *Akkermansia muciniphila*, and an increase in bacteria such as *Prevotella copri* and *Bacteroides vulgatus*, which can synthesize branched-chain amino acids (BCAAs), potentially exacerbating insulin resistance (12–14). However, the relationships among the gut microbiota, metabolic profiles, and unclassified diabetes status remain unexplored, emphasizing the necessity for additional research in this population.

In this investigation, we compared the gut microbiota and metabolic profiles among individuals with UDM, T1DM, T2DM, and healthy controls (HCs), elucidating the intricate relationships between the gut microbiota composition, metabolite modules, and clinical phenotypes across these groups. This comprehensive analysis is intended to explore the features of UDM and unravel potential pathogenic mechanisms, contributing to a more nuanced understanding of diabetes subtypes.

## 2 Methods

### 2.1 Study participants and recruitment

This cross-sectional study included 18 patients with T1DM, 18 patients with UDM, 13 patients with T2DM, and 18 healthy controls (HCs), all of Han descent and under 30 years. We considered the age of onset ≤ 30 as young-onset. The patients were diagnosed according to the World Health Organization guidelines. T1DM was diagnosed based on the presence of acute ketosis or ketoacidosis, the course of insulin replacement therapy, impaired islet function, or positivity for at least one autoantibody (glutamic acid decarboxylase autoantibodies [GADA], insulinoma-associated antigen-2 autoantibodies [IA-2A], or islet cell antibody [ICA]). T2DM was diagnosed based on a typical history of hyperglycemia, no immediate requirement for insulin treatment, and negativity for islet autoantibodies. UDM was diagnosed through an exclusion-based approach, beginning with genetic tests to rule out monogenic diabetes and assessments to exclude secondary causes like infections or pancreatic disorders. The diagnosis also depended on the patient’s clinical profile not matching the criteria for T1DM characterized by autoimmune beta-cell destruction, or T2DM, which involves insulin resistance and relative insulin deficiency. This process ensured the specificity of UDM diagnoses, reserved for cases with unidentified etiologies that do not fit known diabetes types or other defined disorders. All healthy subjects and patients with T2DM tested negative for GADA, IA-2A, and ICA. Additionally, all healthy subjects underwent a standard 75-g oral glucose tolerance test (OGTT) to confirm their normal blood glucose levels. The exclusion criteria for this study included secondary diabetes, acute or chronic inflammatory diseases, infectious diseases, pregnancy, malignant tumors, a history of steroid or immunosuppressive drug use for more than 7 days, a history of treatment with prebiotics, probiotics, antibiotics, or any other medication that could influence the gut microbiota for more than 3 days within the previous 3 months, gastrointestinal diseases, a history of gastrointestinal surgery within the previous year, and hepatic and renal dysfunction. The collected demographic and clinical data included age, sex, diabetes duration, height, weight, body mass index (BMI), systolic blood pressure, and diastolic blood pressure. Additionally, biochemical data such as the 75-g OGTT, C-peptide release test, HbA1c, fasting plasma glucose (FPG), lipid profile, and renal function were collected. All participants provided written informed consent, and this study was approved by the Ruijin Hospital ethics committee.

### 2.2 Metagenomic analysis of the human gut microbiome

Metagenomic sequencing was utilized to investigate the gut microbiome of the four groups in this study. Fecal samples were collected from each patient for metagenomic analysis. Patients had not been treated with antibiotics for at least one month before sampling. Furthermore, they avoided probiotic-rich foods, including yogurt, for a week before collecting the samples. Each sample was immediately frozen at −80°C or temporarily held in personal freezers at −20°C before being transported to the laboratory within a 24-hour window. Total genomic DNA was extracted using the QIAamp Fast DNA Stool Mini Kit from Qiagen, Germany. Paired-end sequencing was performed on the NovaSeq 6000 platform from Illumina, Inc., in San Diego, CA, USA, at Majorbio BioPharm Technology Co., Ltd., in Shanghai, China. Reads that had adapter sequences or low quality, with a length shorter than 50 bp or a quality value lower than 20, were discarded. The remaining reads were aligned to the *Homo sapiens* genome using the NCBI database(GCA_000001405.28) to remove host DNA. The short reads were assembled using Megahit. SOAPaligner was used to map high-quality reads with 95% identity to representative genes, and the abundance of genes in each sample was assessed using RPKM. The overall information of all genes in the environment can be summarized by constructing a non-redundant gene set. This involves clustering the gene sequences predicted from all samples using CD-HIT software(http://www.bioinformatics.org/cd-hit/) with default parameters set to 90% identity and 90% coverage. The longest gene in each cluster is selected as the representative sequence to create the non-redundant gene sets, allowing for the exploration of commonalities and differences among the various samples. The results from this step include a table of gene number and length statistics before and after redundancy removal, as well as the base sequences and amino acid sequences of the genes in the non-redundant gene set. For taxonomic annotations, non-redundant gene sets were aligned to the NR database using DIAMOND software (http://ab.inf.uni-tuebingen.de/software/diamond/) with BLASTP (Version 2.2.28+, http://blast.ncbi.nlm.nih.gov/Blast.cgi). The alignment parameters were set with an expected value (e-value) of 1e-5 (15), employing the best hit approach.

Using the species annotation results from the taxonomic information database corresponding to the NR library, the abundance of each species was calculated by summing the genes associated with that species. The abundance was then assessed at various taxonomic levels, including Domain, Kingdom, Phylum, Class, Order, Family, Genus, and Species for each sample. To construct the abundance table (abundance profile) at the corresponding taxonomic level. At the domain level, 5 were obtained; at the kingdom level, 13; at the phylum level, 149; at the class level, 262; at the order level, 475; at the family level, 848; at the genus level, 2727; and at the species level, 12836.

We used the KEGG database analysis. For KEGG functional annotation, align non-redundant gene set sequences against the gene database (GENES) using BLASTP (BLAST Version 2.2.28+, http://blast.ncbi.nlm.nih.gov/Blast.cgi), set the expected value e-value for BLAST alignment parameter 1e-5. Functional annotation was performed using KOBAS 2.0 (KEGG Orthology Based Annotation System) based on the alignment results (16). The abundances of this functional category were calculated using the sum of the gene abundances corresponding to KO, Pathway, EC, and Module. There are probably 206112 genes that were obtained and annotated.

### 2.3 Nontargeted lipidomic analysis

Human serum samples were analyzed using an ultrahigh-performance liquid chromatography high-resolution mass spectrometry/mass spectrometry (UHPLC-HRMS/MS)-based non-targeted lipidomics platform. Lipidomic analysis was performed using a ThermoFisher Ultimate 3000 UHPLC system coupled to a Q Exactive Orbitrap Mass Spectrometry with a Heated Electrospray Ionization Source. The raw UHPLC-HRMS/MS data were processed using Compound Discoverer (version 3.3, Thermo Fisher) with a lipidomics workflow template. This included retention time alignment, compound detection, and compound group and structural identification of lipids using the LipidBlast library (version 68).

### 2.4 Serum metabolites analysis

The quantification of human serum was performed using the UHPLC-MS/MS platform, which involved several steps, including sample preparation, UHPLC-MS/MS analysis, raw data preprocessing, and the calculation of relative quantification of target metabolites. The metabolites were analyzed using a Thermo Fisher Ultimate 3000 UHPLC system coupled to a Q Exactive Orbitrap Mass Spectrometry in Heated Electrospray Ionization Source with positive and negative modes. The raw data were processed using Xcalibur Software (version 4.0, Thermo Fisher Scientific). In this step, the target metabolites and internal standards were identified, and the integral areas were exported. The relative quantification results were obtained by normalizing the peak areas of the target metabolites to that of the corresponding internal standard.

### 2.5 Statistical analyses

Differences in clinical parameters were analyzed using the chi-square test or Kruskal−Wallis test, and multiple comparisons were corrected using false discovery rate (FDR) *post hoc* tests. To compare metabolite and lipid profiles, orthogonal projections to latent structures discriminant analysis (OPLS-DA) algorithms were used. Variable importance for the projection (VIP) scores were obtained from the OPLS-DA. A *P*_fdr_ < 0.1 was considered statistically significant for metabolites, and a *P* < 0.05, VIP > 1, and fold change > 1.5 were considered statistically significant for lipids. The Kruskal−Wallis rank-sum test was applied to assess the differences in microbial alpha diversity. Permutational multivariate analysis of variance (PERMANOVA) was used to compare microbiota beta diversity, and redundancy analysis (RDA) was used to evaluate the effects of demographic variables on microbiota community variation. The maximum Pearson correlation coefficient between environmental factors and the sample community can be determined using the bioenv function, which identifies the subset of environmental factors corresponding to the highest correlation. RDA is then performed on the sample species distribution table alongside the environmental factors or their selected subset. The significance of the RDA results is assessed through a permutation test similar to ANOVA, focusing on groups where environmental factors influence differences in microbial communities. Significant differences in the relative abundances of taxa were identified using linear discriminant analysis (LDA) effect size (LEfSe) analysis, and *P* values were corrected using the Benjamini and Hochberg FDR. Taxa with LDA values > 2.0 and *P* < 0.05 were considered to be differentially abundant, and taxa with *P*_fdr_ < 0.1 were considered to be significantly different (15). LEfSe (http://galaxy.biobakery.org/) is a software tool designed for discovering high-dimensional biological identifiers and revealing genomic features, including genes, metabolic pathways, and classifications, to distinguish between two or more biological conditions (or taxa). The software first employs the non-parametric factorial Kruskal-Wallis sum-rank test to detect significant differences in abundance, identifying taxa that show marked differences. Next, the Wilcoxon rank-sum test assesses the consistency of these differences across various groups for the identified species. Finally, LEfSe utilizes linear discriminant analysis (LDA) to estimate the impact of the abundance of each component (species, gene, or function) on the observed differential effects.

To determine the effectiveness of the selected features, random forest models were built using microbial features, metabolic features, and a combination of the two types of data to differentiate different groups. We calculated the area under the curve (AUC) for the model using these features, which provided a quantitative measure of the model’s ability to distinguish between UNC and HC. The selected features yielded a maximum AUC, indicating strong discriminatory power. The feature set was refined iteratively based on the ROC analysis results, ensuring that only those features that contributed significantly to the AUC were retained. This process helped us focus on the most relevant predictors while minimizing the potential for overfitting (15).These models were built using the randomForest package in R. The data were analyzed using the Majorbio Cloud Platform (https://cloud.majorbio.com/page/tools/) (17).

## 3 Results

### 3.1 Anthropometric and biochemical measurements of different types of diabetes

**Figure 1:**
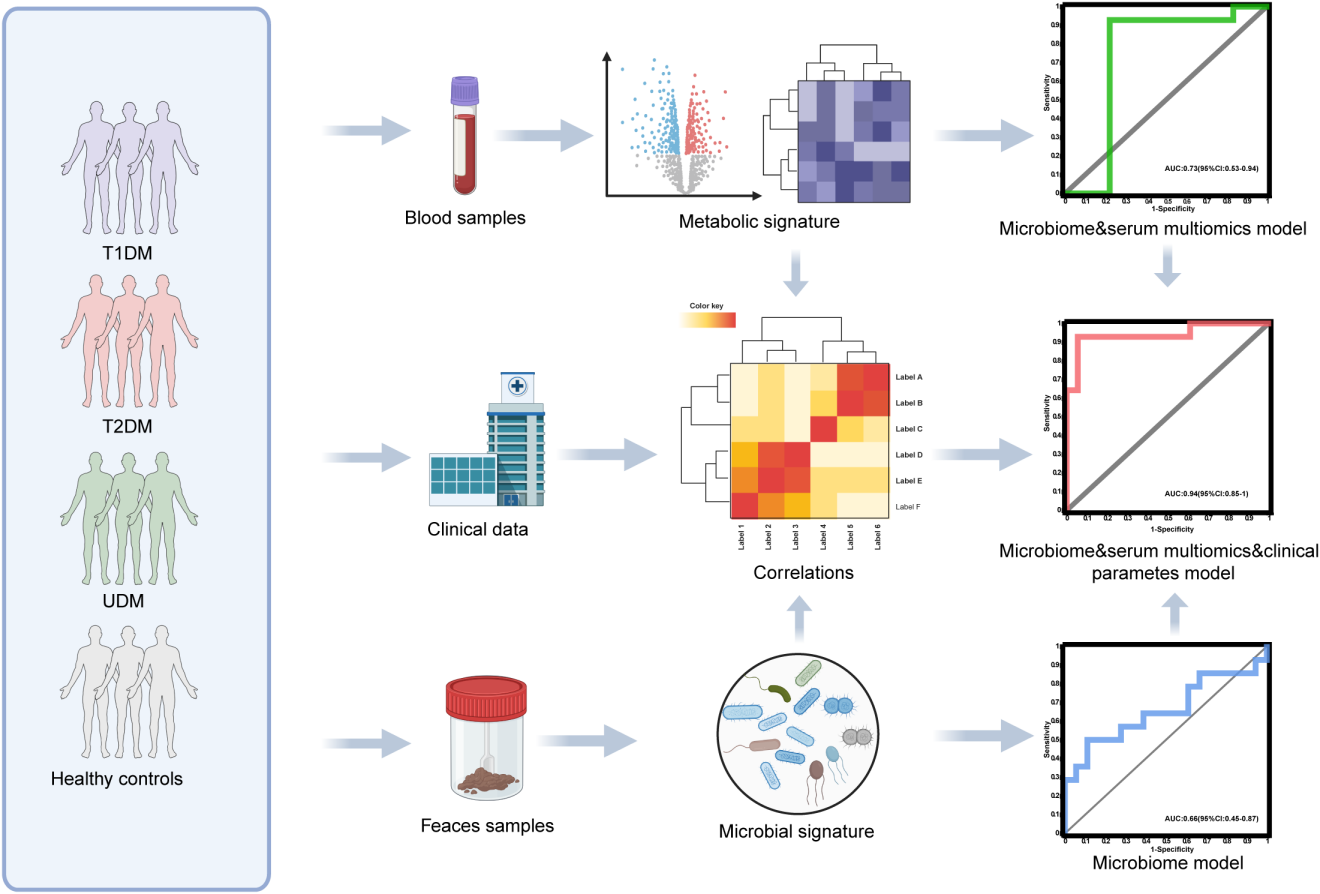
Diagram of the study design. Created with BioRender.com.

The study design is illustrated in **Figure 1**. In this study, participants with T1DM, T2DM, UDM, and HCs under the age of 30 were recruited following a stringent pathological diagnostic and exclusion methodology. The delineation of unclassified diabetes was based on the World Health Organization (WHO) criteria, excluding known types such as T1DM, T2DM, and hybrid diabetes and specific types such as monogenic diabetes, diseases of the exocrine pancreas, endocrine disorders, drug- or chemical-induced diabetes, infections, uncommon specific forms of immune-mediated diabetes, and other conditions discerned through genetic, clinical, and laboratory evaluations. The biochemical characteristics of the study groups are presented in **Table 1**. Elevated fasting plasma glucose (FPG) and hemoglobin A1C (HbA1c) levels were observed across all patient groups relative to those of HCs. Compared with the T1DM group, the UDM group exhibited increased BMI, FPG, and HbA1c, as well as notably increased uric acid (UA) levels. Furthermore, the homeostatic model assessment for insulin resistance (HOMA-IR) was significantly higher in the UDM group than in the HCs and T1DM groups but remained below the T2DM level. The homeostatic model assessment for beta-cell function (HOMA-β) was significantly higher in the HC group compared to both the UDM and T1DM groups, with the UDM group showing higher levels than the T1DM group. Additionally, levels of LDL, HDL, total cholesterol (TC), and triglycerides (TG) were significantly elevated in the UDM group compared to the HCs group. Furthermore, fasting C-peptide (FCP) and postprandial C-peptide (PCP) levels were significantly higher in the UDM group compared to the T1DM group, but significantly lower than those in the T2DM group.

**Table 1:** Baseline anthropometric and biochemical variables.

	Healthy controls (n=18)	Unclassified diabetes (n=18)	T1DM (n=18)	T2DM (n=13)	*P* value between all groups
Age (years)	23.00 (21.00-24.00)	22.00 (16.50-27.00)^#^	22.00 (16.00-26.50)	20.00 (15.00-22.00)	0.963
Age of onset (years)	/	22.00 (15.75-26.25)	21.50 (15.75-26.25)	19.00 (18.00-25.00)	0.931
Male/Female (n)	8/10	10/8	6/12	7/6	0.54
BMI (kg/m^2^)	19.85 (19.27-21.55)	26.95 (24.48-30.26)^*^	21.06 (19.28-25.34)	26.37 (23.38-28.45)*	<0.001
FBG (mmol/L)	4.73 (4.46-4.96)	7.52 (6.19-10.30)^*^	6.04 (5.49-11.64)*	7.20 (6.94-12.00)*	<0.001
PBG (mmpl/L)	5.85 (4.60-6.81)	15.97 (8.78-18.78)^*^	16.44 (8.67-25.66)*	14.18 (7.14-17.51)*	<0.001
HbA1c (%)	5.05 (4.67-5.12)	9.65 (7.72-10.47)^*^^	8.40 (6.70-10.60)*	6.00 (5.20-9.80)*	<0.001
FCP (ng/ml)	1.61 (1.41-2.33)	1.90 (1.68-2.57)^#^^	0.11 (0.01-0.49)*	3.83 (1.97-3.92)*	<0.001
PCP (ng/ml)	6.92 (5.54-10.89)	4.53 (2.98-5.28)^*#^^	0.14 (0.01-2.44)*	10.12 (5.98-39.82)	<0.001
TC (mg/dL)	3.90 (3.32-4.38)	4.75 (3.74-5.21)^*^	4.32 (3.96-4.59)	5.17 (3.67-5.49)	0.054
TG (mg/dL)	0.62 (0.56-0.99)	1.40 (0.80-1.89)^*#^	0.85 (0.69-1.05)	2.08 (1.29-2.55)	<0.001
HDL (mg/dL)	1.38 (1.30-1.57)	1.12 (0.90-1.46)^*#^	1.55 (1.23-1.71)	1.02 (0.91-1.07)*	<0.001
LDL (mg/dL)	1.98 (1.68-2.51)	2.66 (1.99-3.75)^*^	2.52 (2.12-2.90)*	3.19 (2.80-3.56)*	<0.001
UA (μmol/L)	313.00 (255.75-369.00)	362.50 (274.00-445.00)^#^	286.00 (230.00-345.50)	374.00 (362.00-481.00)	0.025
HOMA-IR	1.33 (0.93-1.65)	2.63 (2.13-5.91)^*#^^	0.41 (0.11-0.86)*	4.94 (3.10-7.90)*	<0.001
HOMA-β	101.72 (90.18-119.19)	58.19 (29.98-85.25)^*#^	6.81 (1.46-15.21)*	60.46 (23.88-133.56)	<0.001

The data are presented as the median (25th–75th percentile). *versus healthy controls, P < 0.05; ^#^versus T1DM patients, P < 0.05; ^versus T2DM patients, P < 0.05. T1DM, type 1 diabetes mellitus; T2DM, type 2 diabetes mellitus; BMI, body mass index; FBG, fasting blood glucose; PBG, postprandial blood glucose; HbA1c, hemoglobin A1c; FCP, fasting C-peptide; PCP, postprandial C-peptide; TC, cholesterol; TG, triglyceride; HDL, high-density lipoprotein; LDL, low-density lipoprotein; UA, uric acid; HOMA-IR, homeostasis model assessment for insulin resistance; HOMA-β, homeostasis model assessment-β.

### 3.2 Structural modulation of the gut microbiota in the four groups

**Figure 2:**
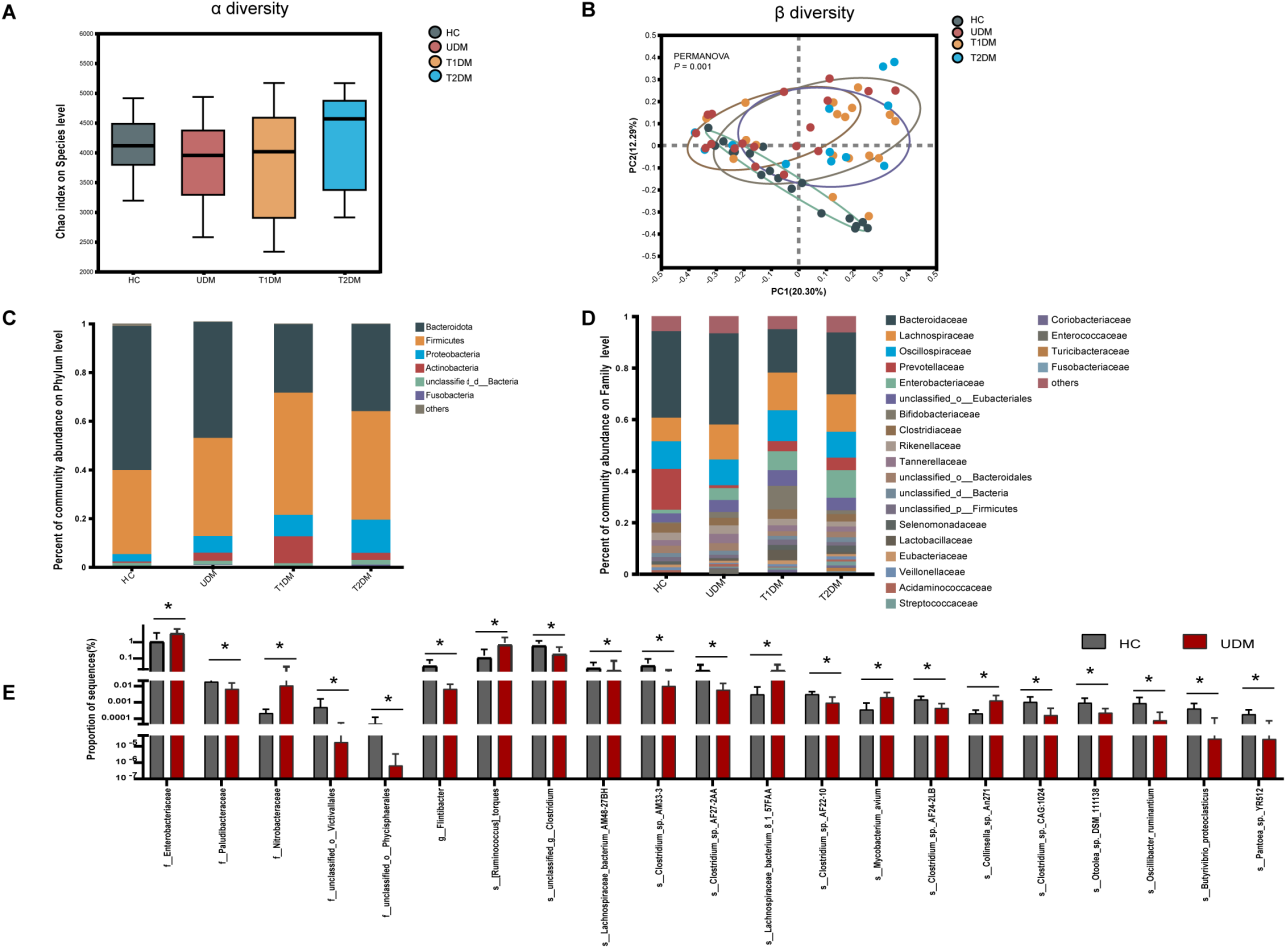
The structural shifts and signatures of the gut microbiota in the four groups. **(A)**. Microbial community richness and diversity (Chao 1 index; *P* = 0.3967). **(B)**. Principal coordinate analysis (PCoA) analysis based on PERMANOVA (*P* = 0.001). **(C, D)**. The relative abundance of microbial taxa at the phylum and family levels; phyla or genera with a relative abundance <1% in each sample were merged into others. **(E)**. Bar charts showing the relative abundance of taxa that were exclusively altered in patients with UDM compared with HCs. HCs, healthy controls; T1D, type 1 diabetes; T2D, type 2 diabetes; UDM, unclassified diabetes; PCoA, principal coordinate analysis; PERMANOVA, permutational multivariate analysis of variance. Bar charts show the mean ± SD. *P_fdr_ < 0.1.(HCs,n=18:UDM,n=18;T1DM,n=18;T2DM,n=13).

First, we analyzed the microbial diversity of the four groups. The Chao index results indicated no significant difference in bacterial richness across the groups(healthy controls: 4118 ± 504.9; T1DM patients: 3825 ± 828.7; T2DM patients: 4225 ± 828.1; UDM patients: 3847 ± 688.9; P > 0.05)(**Figure 2A**). Similarly, the Shannon and Simpson indices also demonstrated no significant differences in bacterial richness among the groups (**Supplementary Figure S1**). Principal coordinate analysis (PCoA) based on the Bray–Curtis distance revealed significant differences in the overall microbial features across the four groups (PERMANOVA, *P* = 0.001) (**Figure 2B**). The bacterial community structure in young-onset UDM patients was significantly distinct from that in HC and T1DM patients (PERMANOVA, T1DM vs HC: *P* = 0.005; T2DM vs HC: *P* = 0.005; UDM vs HC: *P* = 0.001; T1DM vs UDM: *P* = 0.019), underscoring the different microbial composition associated with UDM.

### 3.3 Taxonomic changes in microbial composition in young-onset UDM patients

Next, we analyzed the microbial composition at different taxonomic levels, with the phylum and family compositions shown in **Figures 2C, D**. LEfSe analysis revealed no distinct bacterial phylum structure unique to UDM at the phylum and family levels*. Bacteroidetes* and *Firmicutes* dominated across all groups, followed by *Proteobacteria* and *Actinobacteria*. Significantly, patients with UDM had increased levels of Actinobacteria (0.5636% in HC vs. 3.331% in UDM; *P*=0.0005314, FDR adjusted=0.03154) and Proteobacteria (3.057% in HC vs. 6.869% in UDM; *P*=0.001644, FDR-adjusted=0.0515) compared with HC.

LEfSe analysis was employed to discern differentially abundant microbial species among HCs, T1DM patients, T2DM patients, and UDM patients. A total of 81, 34, and 190 species were identified as differentially abundant between T1DM and UDM, T2DM and UDM, and UDM and healthy controls (HCs), respectively(LDA value > 2, *P* < 0.05) (**Supplementary Tables S1-S3**). The results from the LEfSe analysis indicate that the UDM group has the fewest differential microbial populations compared to the T2DM group. To determine the potential influence of host factors on microbial composition, we conducted a redundancy analysis (RDA) to ascertain potential confounders within these groups. Key host factors, including age, sex, BMI, and diabetes duration, were integrated into the RDA model. Our analysis revealed that, even after adjusting for these confounding factors, 21 taxa in young-onset UDM patients exhibited significant differential abundance compared to healthy controls (HCs) (LDA value > 2, *P_fdr_* < 0.1). Among these, 6 taxa were particularly enriched in UDM patients, including *Lachnospiraceae* and *Enterobacteriaceae*. These taxa are associated with metabolites involved in carbohydrate and amino acid metabolism, suggesting a disturbed gut microbiome’s involvement in carbohydrate and amino acid metabolic pathways (18), while there was a notable depletion of 15 species, such as *Flintibacter, Butyrivibrio_proteoclasticus*, *s_Clostridium_sp_AF27_2AA* and *s_Clostridium_sp_AM33_3* (**Figure 2E**). Additionally, we identified critical functional alterations in the gut microbiota of young-onset UDM patients. There was a significant enrichment of carbon metabolism pathways in these individuals compared to healthy controls, indicating a distinctive metabolic signature. Moreover, the amino sugar and nucleotide sugar metabolism pathways were also significantly enriched in UDM patients compared to those in T1DM and T2DM patients. These findings suggest that the gut microbiota may be involved in the pathogenesis of UDM and shed light on metabolic dysregulation in this disease (**Supplementary Figure S2**).

### 3.4 Associations of the microbiota with serum metabolites and lipids

**Figure 3:**
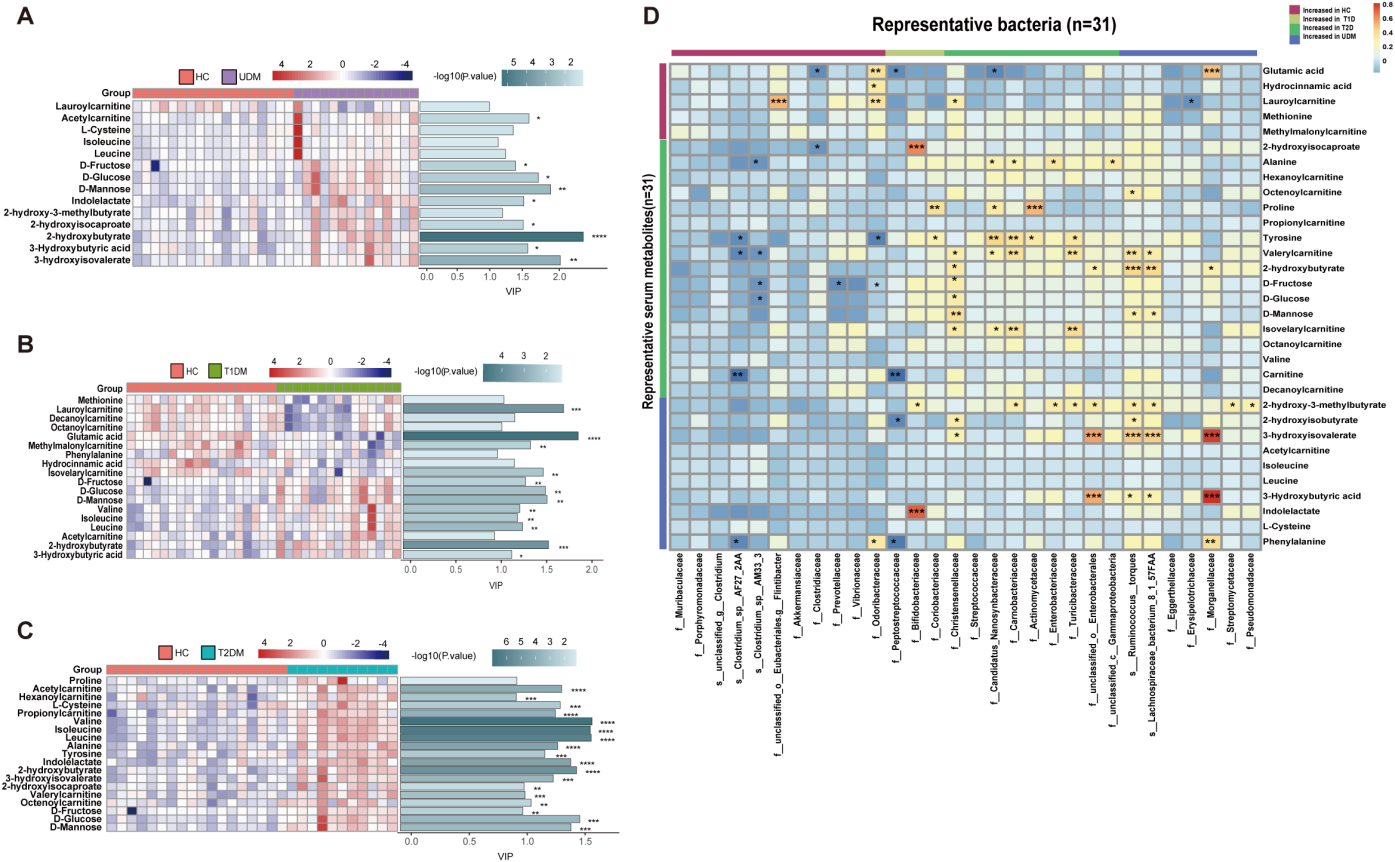
Differentially abundant serum metabolites and their associations with gut bacteria. **(A–C)**. Distribution of serum-enriched differentially abundant metabolites between the UDM group and the HC, T1DM, or T2DM group. Variable importance for the projection (VIP) scores were obtained via OPLS-DA. **(D)**. Associations of representative bacteria and serum metabolites that were altered in UDM patients, T1D patients, T2D patients, or both compared with HCs were assessed by Spearman’s correlation analysis. *P<0.05, **P<0.01, ***P<0.001, ****P<0.0001.

**Figure 4:**
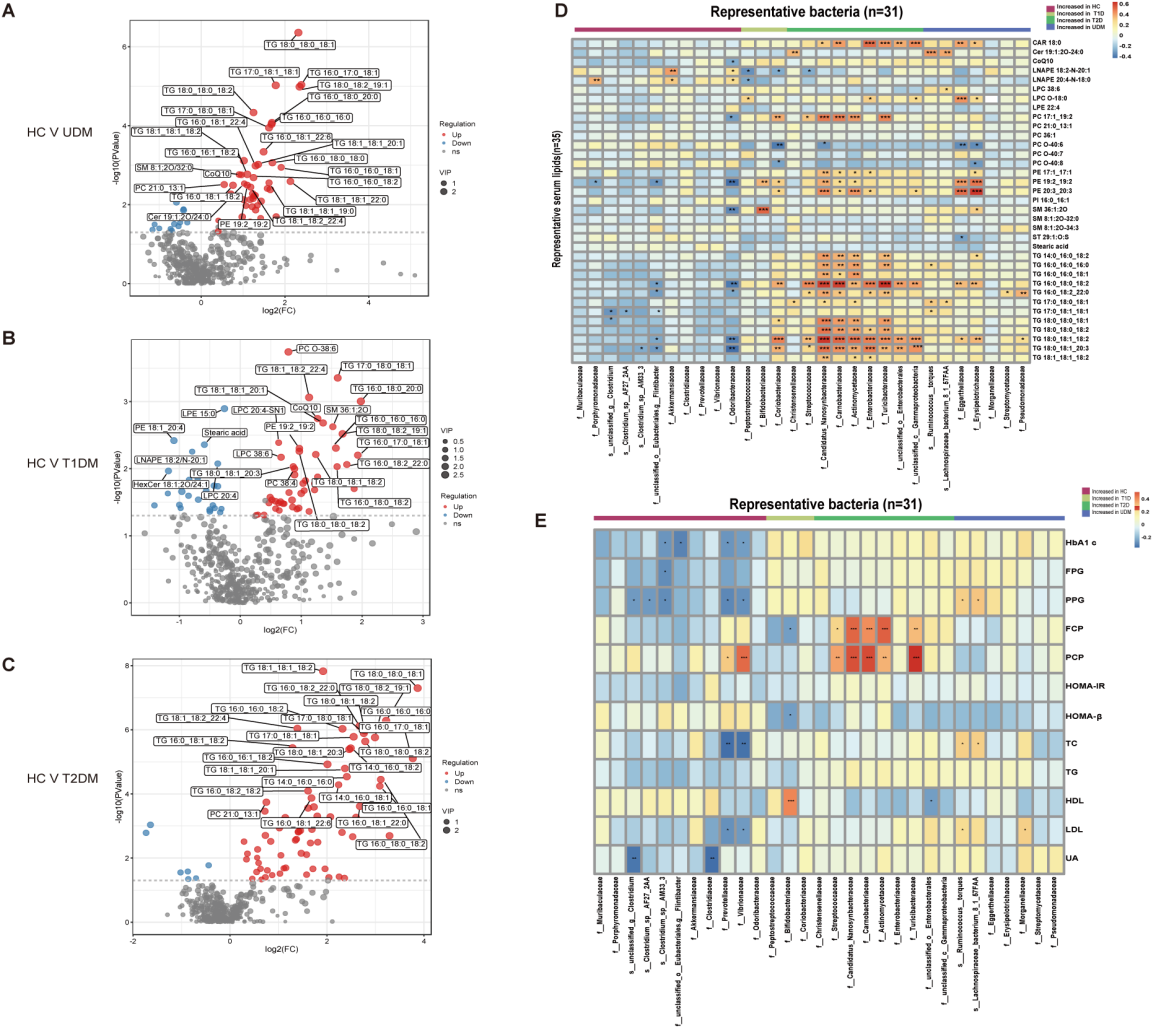
Differentially abundant serum lipids and their associations with gut bacteria. **(A–C)** Volcano plots demonstrating differential lipids between HCs and UDM, T1DM, or T2DM patients. p<0.05, VIP>1, and FC>1.5 were used to screen for differentially abundant serum lipids. **(D)** Associations of representative bacteria and serum lipids that were altered in UDM patients, T1D patients, T2D patients, or both compared with HCs were assessed by Spearman’s correlation analysis. **(E)** Associations of differentially abundant taxa and clinical parameters in patients. *Pfdr < 0.1, **Pfdr < 0.05, ***Pfdr < 0.01, ****Pfdr < 0.001. FC, fold change; OPLS-DA, orthogonal partial least squares discriminant analysis; VIP, variable influence on projection; HC, healthy controls; T1D, type 1 diabetes; T2D, type 2 diabetes; UDM, unclassified diabetes.

We observed significant differences in serum metabolites between patients with diabetes and HCs (**Supplementary Figure S3**). Specifically, the numbers of enriched differentially abundant metabolites in the UDM, T1DM, and T2DM groups compared to those in the HC group were 9, 12, and 18, respectively (*P_fdr_* <0.1) (**Figures 3A-C**). Notably, metabolites such as indolelactate, 3-hydroxyisovalerate, acetylcamitine, and 2-hydroxyisocaproate were more abundant in the UDM and T2DM groups than in the HC group. Additionally, we analyzed serum lipids and revealed significant differences across the groups (**Supplementary Figure S4**), highlighting the metabolic distinctions inherent to diabetes. In patients with UDM, we identified 50 differential lipids, predominantly triglycerides (TGs), which were increased (**Figures 4A-C**). Subsequent correlation analysis explored the relationships between differentially abundant bacteria and metabolites. This revealed that bacteria enriched in HCs had a strong positive correlation with HC-enriched metabolites but exhibited a negative correlation with diabetes-enriched metabolites (**Figure 3D**). Notably, a decrease in carnitine and its derivatives, such as valerylcarnitine and lauroylcarnitine, was observed in UDM patients. These compounds are known to enhance glucose utilization, improve lipid profiles, and reduce oxidative stress markers, suggesting that their protective effects may be diminished in UDM (**Figure 3D**) (19). In individuals with young-onset UDM, an increase in specific metabolic markers, including 3-hydroxybutyric acid, BCAAs, and their catabolic intermediates, was noted. These markers have been associated with an increased risk of transitioning from normoalbuminuria to macroalbuminuria and CKD (20–22). The abundances of bacteria such as *s_Ruminococcus_torques* and *s_Lachnospiraceae_bacterium_8_1_57FAA* were positively correlated with the abundances of metabolites such as 3-hydroxyisovalerate and 3-hydroxybutyric acid, suggesting an increased likelihood of complex diabetic nephropathy in UDM patients. We also observed that amino acids, fatty acid derivatives, and organic acids were enriched in the T2DM group, including alanine, valerylcarnitine, and 2-hydroxyisocaproate, which have been reported to be associated with abnormal fat metabolism and insulin resistance (6, 23, 24).TG and PE were enriched in the UDM and T2DM groups. Additionally, a strong positive correlation between bacteria and lipids (TG and PE) in the UDM and T2DM groups indicates potential parallels in their pathogenic processes (**Figure 4D**).

### 3.5 Associations of the altered microbes and metabolites with clinical parameters

To understand the role of the gut microbiota in the progression of diabetes, we analyzed the associations between clinical parameters and differentially abundant bacteria or metabolites in the four groups. We discovered that certain taxa related to young-onset T2DM, including *Streptococcaceae* (25) and *Actinomycetaceae* (26), were significantly correlated with glucose metabolism and pancreatic beta cell function, corroborating previous findings. These taxa had a positive correlation with PCP and FCP (**Figure 4E**). In the UDM cohort, bacteria such as *Ruminococcus:torques*, *Lachnospiraceae_bacterium_8_1_57FAA*, and Nitrobacteraceae were positively associated with PBG and FCP. Furthermore, we discovered novel associations between young-onset UDM and increased metabolites such as 2-hydroxy-3-methylbutyrate, 2-hydroxyisobutyraten, and 3-hydroxyisovalerate, which are all positively correlated with PBG, FBG, and FCP. Particularly in UDM, high levels of 3-hydroxyisovalerate, 3-hydroxyisobutyrate, and phenylalanine were strongly related to blood uric acid, indicating their potential role in renal function, suggesting a heightened risk of diabetic nephropathy in UDM patients. In UDM, we observed an enrichment of metabolites integral to amino acid metabolism, including 2-hydroxy-3-methylbutyric acid, cysteine, and phenylalanine. This enrichment aligns with an increase in bacterial pathways for amino sugar metabolites, providing insight into the metabolic landscape of UDM. In T2DM patients, elevated levels of D-fructose, D-glucose, and D-mannose were linked to key glucose and lipid metabolism parameters (**Supplementary Figure S5A**). Moreover, TG, which was significantly elevated in T2DM patients, correlated strongly with glucose metabolism (**Supplementary Figure S5B**). Correlations analysis indicates potential links between the gut microbiota, metabolites, and clinical parameters in UDM (**Supplementary Figure S6**). The results from the LEfSe analysis indicate that the UDM group has the fewest differential microbial populations compared to the T2DM group. A total of 81, 34, and 190 species were differentially abundant between T1DM and UDM, T2DM and UDM, and UDM and HCs, respectively (LDA value > 2, P < 0.05) (**Supplementary Tables S1-S3**). Meanwhile, the analysis of alterations in gut microbiota functionality revealed that the differential pathways between the UDM and T2DM groups were relatively fewer compared to the other two groups (**Supplementary Figure S2**). Regarding the differential metabolites compared to the HC group, the types of differential metabolites in UDM and T2DM were more similar, primarily consisting of TG and amino acid metabolites, and showed a positive correlation with their respective enriched microbial communities (**Figures 3D**, **4D**). Therefore, the gut microbiota structure of patients with unclassified diabetes is relatively similar to that of patients with type 2 diabetes.

### 3.6 Multiomic classifier discriminating patients with young-onset UDM from patients in the other three groups

**Figure 5:**
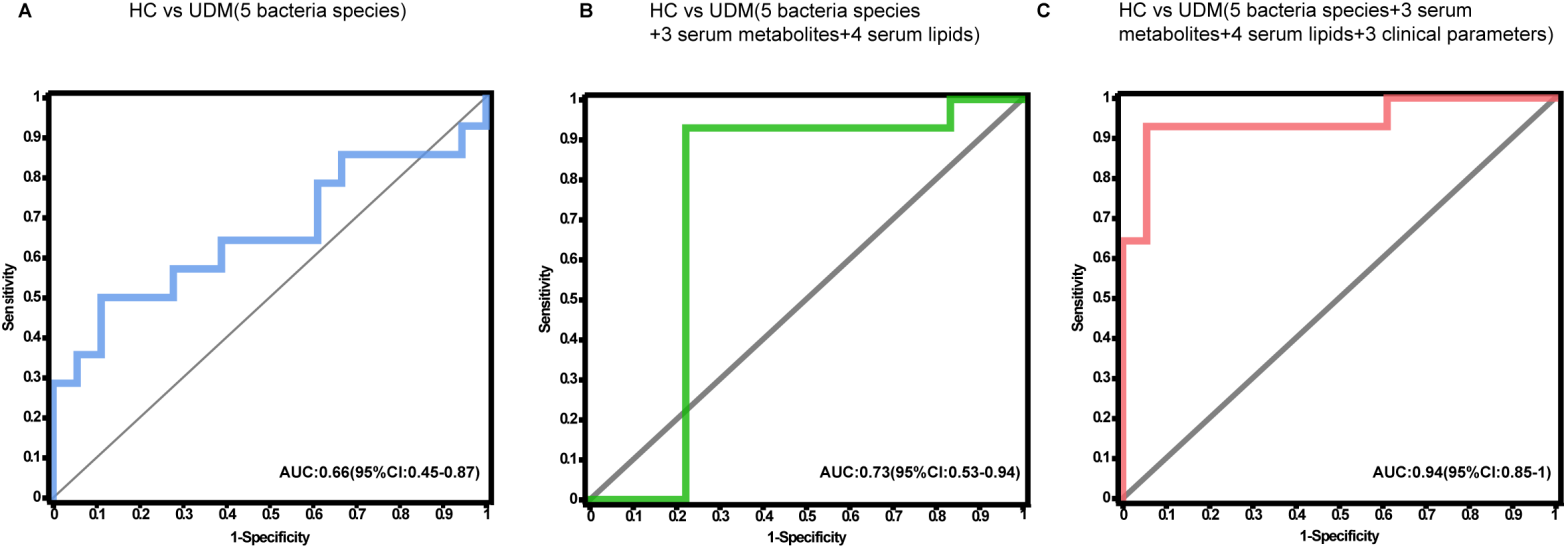
Disease classification based on the signatures of the gut microbiome and metabolome. Random forest classifiers composed of bacteria **(A)**, combinations of metabolites **(B)**, and clinical parameters **(C)** were constructed to discriminate patients with UDM from HCs. HC, healthy controls; UDM, unclassified diabetes; AUC, area under the curve.

To ascertain the potential of the gut microbiota and metabolites as biomarkers for the differential diagnosis of diabetes, we constructed random forest models based on changes in fecal taxonomic or metabolic features between HCs and UDM patients (**Supplementary Table S4**). The model revealed a bacterial signature of 5 distinct species that could differentiate UDM patients from HCs, with an area under the curve (AUC) of 0.66 (95% CI 0.45–0.87) (**Figure 5A**). An additional random forest model was assessed for its diagnostic efficacy utilizing a combination of 7 serum biomarkers, including 3 metabolites and 4 lipids. Notably, this model produced an AUC of 0.73 (95% CI 0.53–0.94) for distinguishing patients with young-onset UDM from HCs (**Figure 5B**). Further enhancement of the model with a panel of five bacterial species, seven serum biomarkers, and three clinical parameters increased its discriminative power, yielding an AUC of 0.94 (95% CI 0.85–1) in differentiating UDM patients from HCs (**Figure 5C**), demonstrating the potential of this comprehensive approach for accurate diagnosis.

## 4 Discussion

Eason RJ et al. (27) demonstrated that adults diagnosed with type 1 diabetes who are negative for islet antibodies have genetic and C-peptide characteristics that are intermediate between those of type 1 and type 2 diabetes. This suggests a significant misclassification within this cohort, potentially including individuals with islet antibody-negative autoimmune (type 1) diabetes as well as those with nonautoimmune (predominantly type 2) diabetes who have been erroneously classified. Such misclassification can lead to inappropriate treatment regimens, including unnecessary lifelong insulin therapy, and hinder access to effective type 2 diabetes treatments.

Currently, the high prevalence of type 2 diabetes in adults makes robustly discriminating true type 1 diabetes from atypical presentations of type 2 diabetes challenging. Some reported characteristics of type 1 diabetes in older adults, such as low islet autoantibody prevalence, may reflect the inadvertent study of those with and without autoimmune diabetes, and some research in this area suggests a need to combine clinical diagnosis with gut microbiota and metabolite profile tests in this setting (28–30).

The World Health Organization (WHO) introduced UDM in 2019 when there was no clear diagnostic category (4). In this study, we revealed that unclassified diabetes patients have different gut microbiota and metabolite profiles than healthy individuals as well as classic T1DM and T2DM patients. Remarkably, the gut microbiota of unclassified diabetes patients displayed distinctive characteristics, with significantly increased abundances of *s:_Ruminococcus:torquess* and *Lachnospiraceae_bacterium_8_1_57FAA* and decreased abundances of *s:unclassified_g:Clostridium*, *s:Clostridium_sp:AF27_2AA* and *s:Clostridium_sp:AM33_3* compared with those in the other groups. There was a clear correlation among the gut microbiota, serum metabolites, and clinical phenotypes. Furthermore, the gut bacterial pathway of “Amino sugar and nucleotide sugar metabolism” was significantly enriched in young-onset UDM patients, differentiating them from T1DM and T2DM patients and suggesting that unique metabolic processes are involved in UDM.

In patients with unclassified diabetes, we detected an enrichment of branched-chain amino acids (BCAAs) and their derivatives in the blood, which correlated with glucose and lipid metabolism. Large human population studies have shown that a high intake of dietary BCAAs increases the risk of T2DM (31). In our study, BCAAs and their derivatives might affect glucose metabolism and sensitivity in patients with unclassified diabetes, which was consistent with the functional differences in the bacteria. Metabolites such as indolelactate, 3-hydroxyisovalerate, acetylcarnitine, and 2-hydroxyisocaproate were found at higher concentrations in the UDM and T2DM groups compared to the HC group, and these metabolites are positively associated with the risk of developing T2DM (32–34). In our population, most UDM and T2DM patients exhibited both obesity and deteriorated lipid metabolism, suggesting that these elevated metabolite levels may be linked to the shared obesity characteristics in both groups. Indeed, Patients with UDM or T2DM had We found that, serologically, UDM was more similar to T2DM, but T2DM was dominated by TG enrichment and UDM by amino acid derivatives. Moreover, high levels of 3-hydroxyisovalerate and 3-hydroxyisobutyrate were strongly related to blood uric acid in the UDM group, which could suggest that unclassified diabetes patients had a poor renal function in the subsequent course. Therefore, this finding suggests that patients with unclassified diabetes mellitus need to pay attention to changes in renal function in later follow-up.

Importantly, we developed a prediction model for UDM based on gut microbial signatures and metabolic features, which demonstrated high accuracy in distinguishing patients with this disease from HCs. Furthermore, we have shown that the predictive power of the model can be enhanced by incorporating metabolites, and the utilization of the “5 + 7+3” model enables simultaneous differentiation of patients with UDM from HCs. The differential metabolite composition between the UDM and HC groups was similar to that of the T2DM and HC groups. However, the increasing prevalence of obesity among patients with T1DM due to environmental and lifestyle factors, the presence of ketosis-prone individuals in patients with T2DM and idiopathic T1DM, and the unavailability of autoantibody detection facilities in certain clinics pose challenges in accurately classifying different types of diabetes. In this regard, comprehending the metabolic and microbiota characteristics of unclassified diabetes mellitus patients is crucial for gaining insights into disease pathogenesis and prognosis.

Although our study provides valuable insights into unclassified diabetes, it has several limitations that should be considered. First, the cross-sectional design of our study cannot establish a causal relationship between the identified gut microbiota and young-onset unclassified diabetes. Additionally, the sample size is restricted due to the limited number of adolescent-onset diabetes cases we were able to collect. In future research, we plan to continue gathering data on adolescent cases of type 1, type 2, and unclassified diabetes to develop classification models that can differentiate UNC from other diabetes types based on gut microbiota and metabolites. Moreover, the relatively small sample size and the restriction of subjects to a specific ethnic population and geographic region may limit the generalizability of our results. The taxonomy-based microbiome analysis heavily relies on existing databases for taxonomic assignment in our manuscript. This dependence can introduce biases if the database is incomplete or not representative of the microbial diversity present in the samples.

Finally, despite our efforts to address confounding factors when comparing the three groups (sex- and age-matched patients with comparable demographic characteristics, antibiotic exposure, and comorbidities), our findings could be influenced by other confounders, such as disease duration and dietary intake. Therefore, the significance of these findings should be confirmed through larger prospective follow-up studies involving more diverse ethnic populations and geographic regions.

## 5 Conclusion

Our study revealed distinct characteristics of the gut microbiota and metabolic profiles in patients with unclassified diabetes, distinguishing them from healthy individuals. Additionally, we observed correlations between these profiles and aspects of glucose metabolism and islet function, suggesting their potential involvement in the development and progression of unclassified diabetes. Importantly, we also found that patients with unclassified diabetes may experience impaired renal function in the future, highlighting the need for careful monitoring. Overall, the findings from this study provide valuable insights that could contribute to the classification and comprehension of diabetes through the identification of novel pathways.

## Data Availability

The datasets presented in this study can be found in online repositories. The names of the repository/repositories and accession number(s) can be found in the article/**Supplementary Material**.
