# Supplementary material for: Gut microbiota and metabolic profiles in adults with unclassified diabetes: a cross-sectional study
Source: Front Endocrinol (Lausanne). 2024 Nov 11;15:1440984. doi: 10.3389/fendo.2024.1440984 (PMC11586653; doi:10.3389/fendo.2024.1440984)
Supplement: Supplementary file 1 [file DataSheet1.docx]

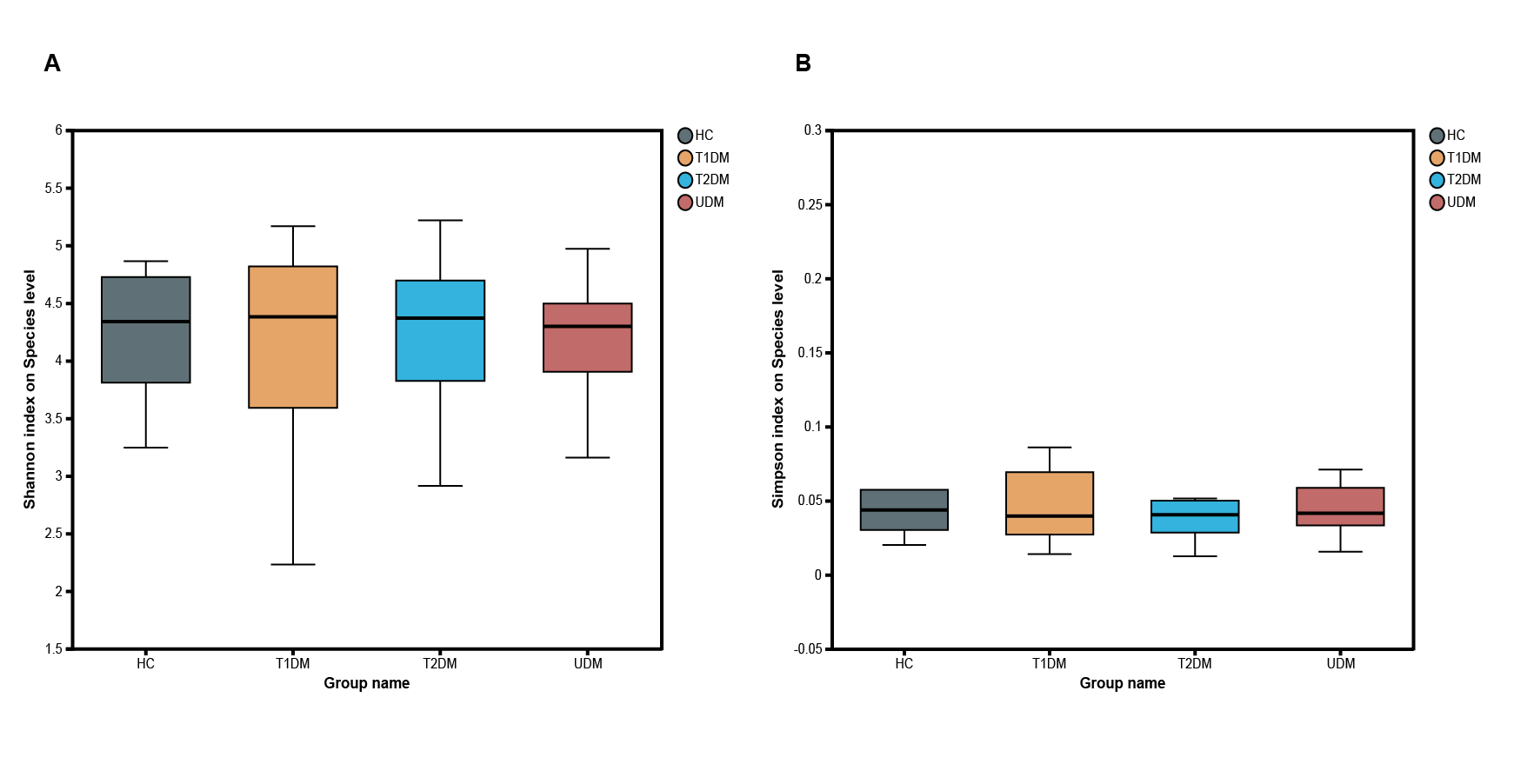


Supplementary Figure 1. The microbial community richness and diversity between groups (Shannon index and Simpson index).


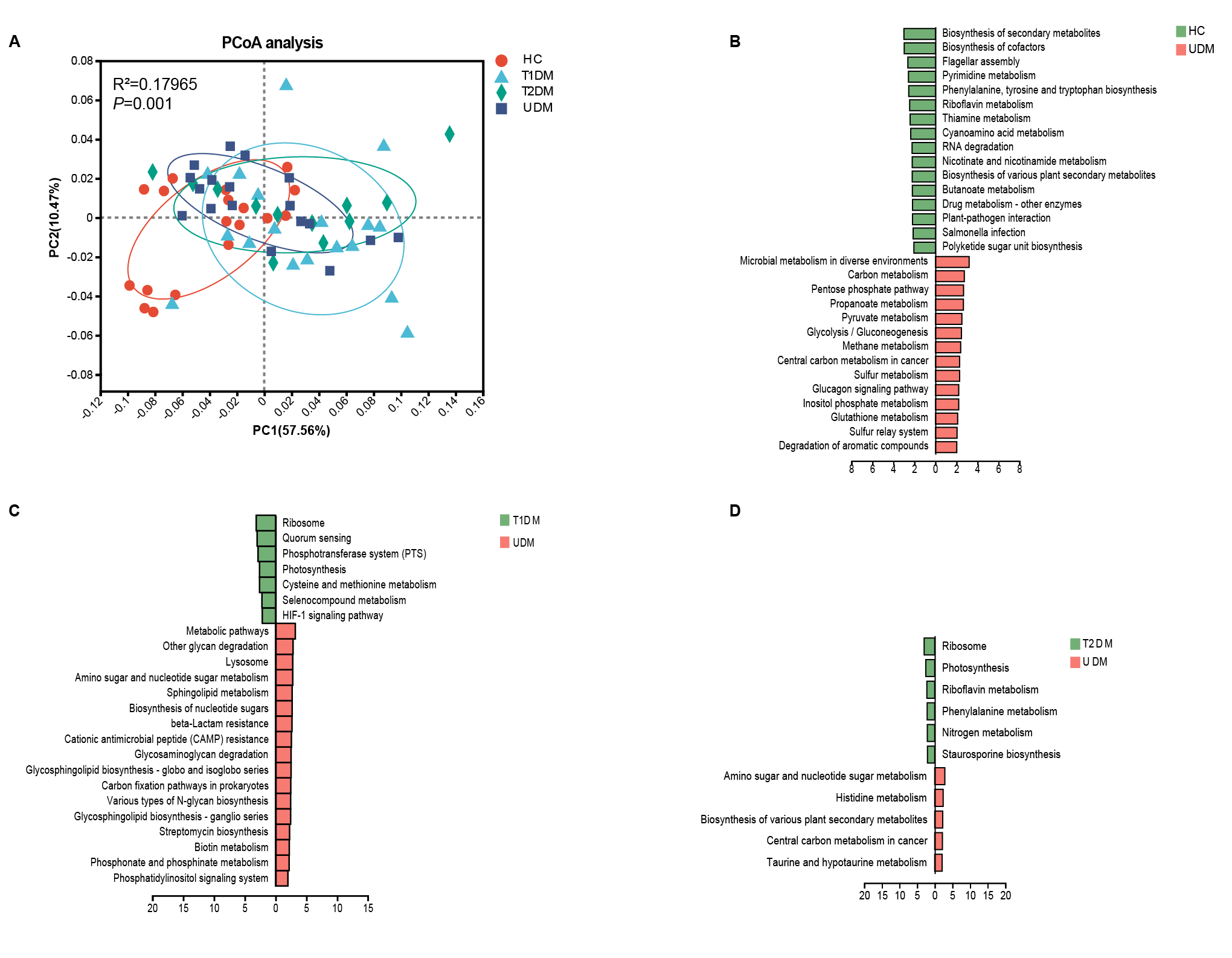


Supplementary Figure 2. Alterations in gut microbiota functionality.

A. Principal coordinate analysis (PCoA) analysis based on PERMANOVA (p = 0.001). B-D. KEGG pathway analysis of differentially abundant gut microbiota between patients with adult-onset UDM and HC (B), between patients with adult-onset T1DM and UDM (C), and between patients with adult-onset T2DM and UDM (D). Pathways with LDA value > 2 and P < 0.05 were considered differentially abundant. KEGG, Kyoto Encyclopedia of Genes and Genomes; HC, healthy control; T1DM, type 1 diabetes mellitus; T2DM, type 2 diabetes mellitus; UDM, unclassified diabetes; LDA, linear discriminant analysis.


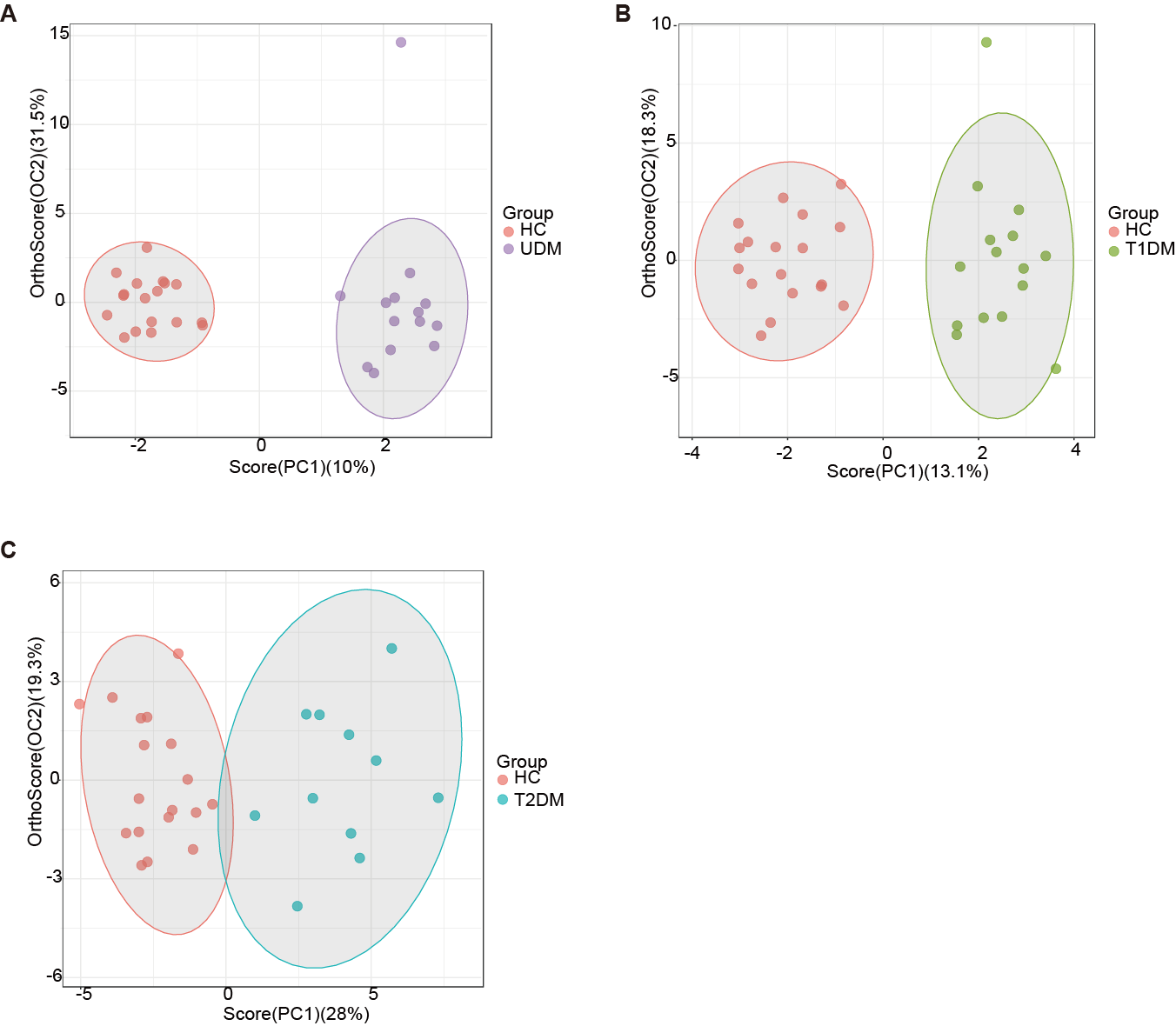


Supplementary Figure 3. Profiles of serum metabolites.

Profiles of serum metabolites between HC and UDM(A) or T1DM(B) or T2DM group(C).


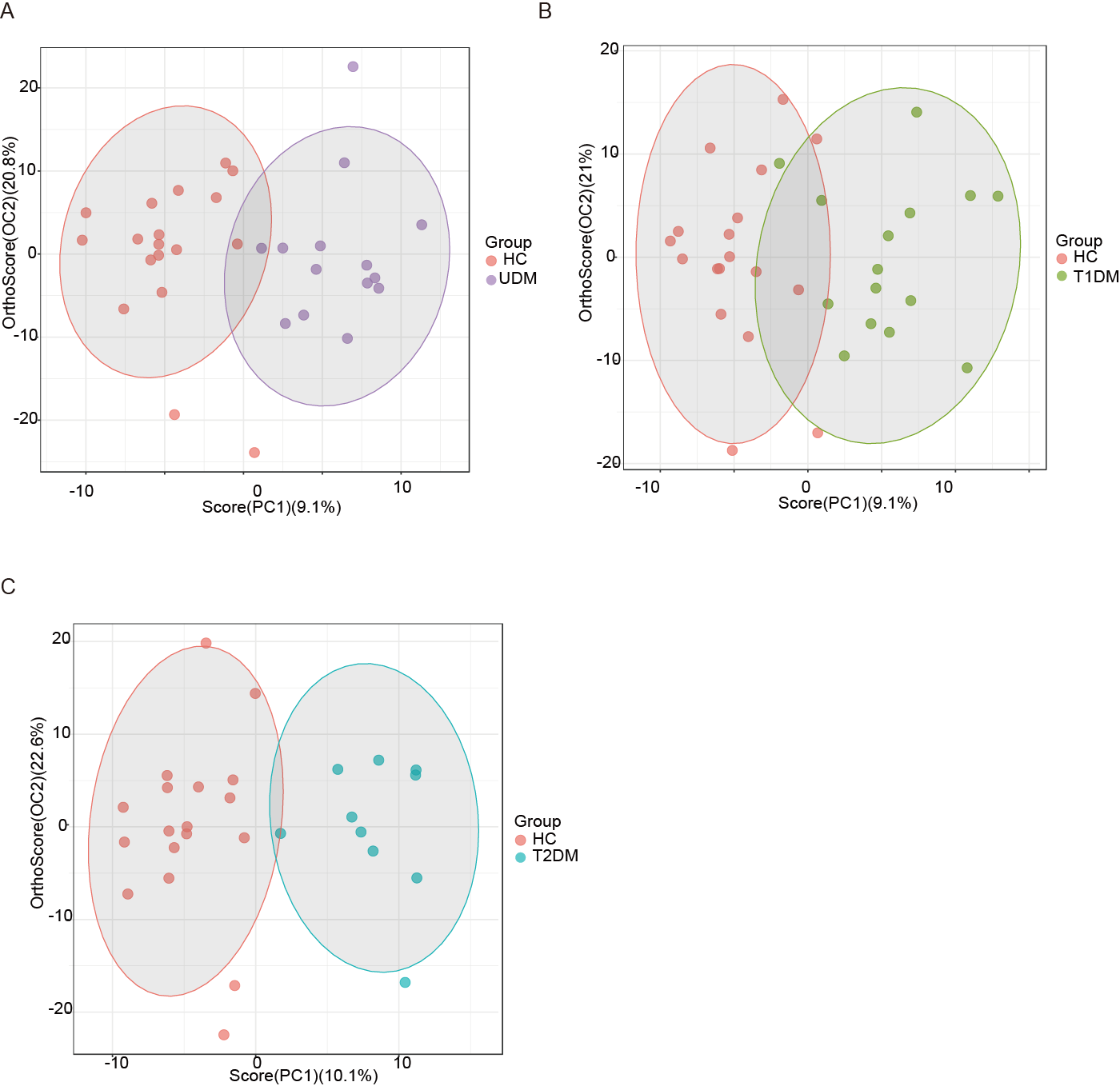


Supplementary Figure 4. Profiles of lipids.

Profiles of lipids between HC and UDM(A) or T1DM(B) or T2DM(C) group.


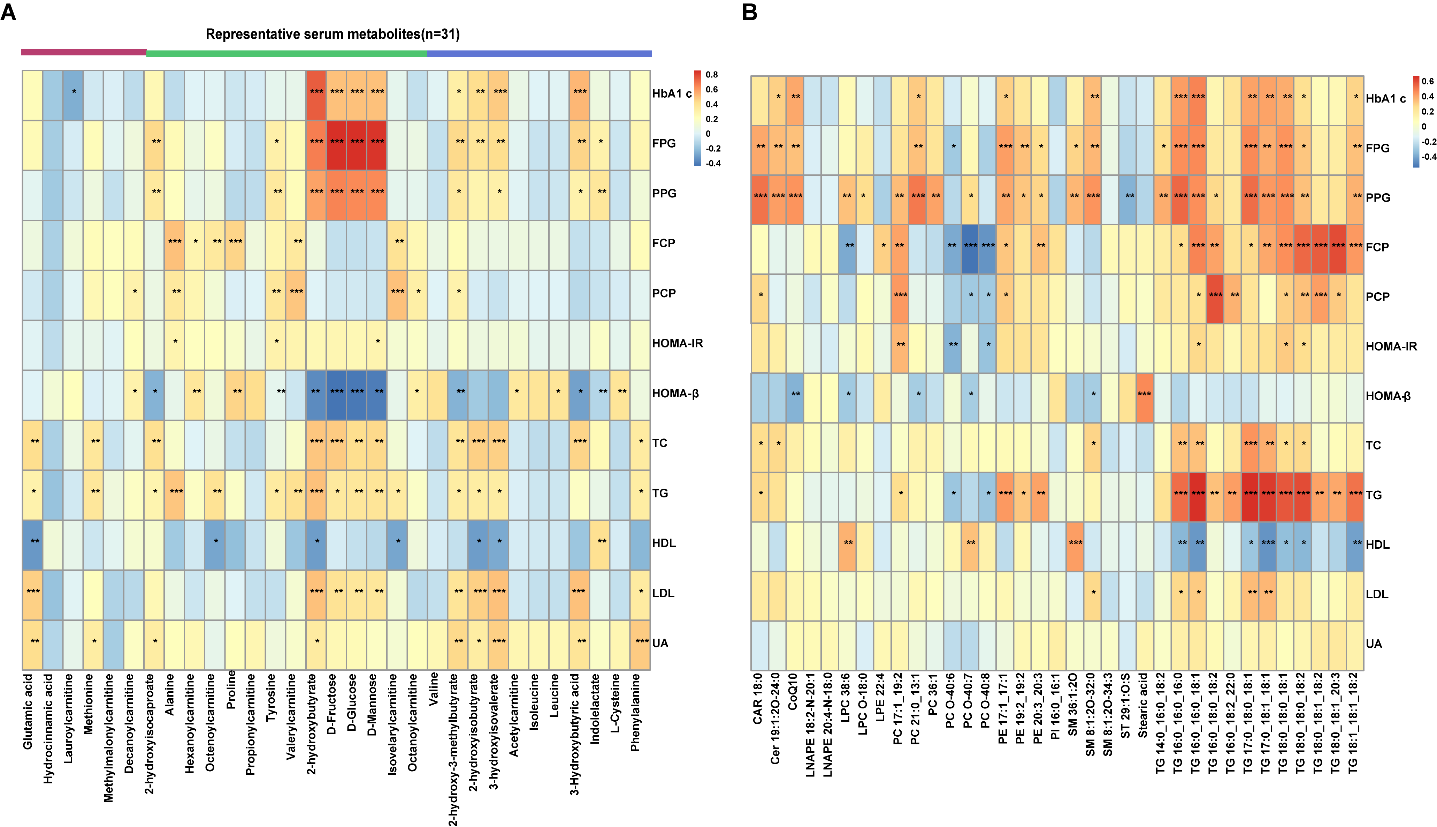


Supplementary Figure 5. Association analysis of serum metabolites, lipids, and clinical parameters.

Associations of serum metabolites (A) and serum lipids(B) and clinical parameters in patients. Correlations were calculated with Spearman’s correlation analysis. FBG, fasting blood glucose; PBG, postprandial blood glucose; HbA1c, hemoglobin A1c; FCP, fasting C-peptide; PCP, postprandial C-peptide; HOMA2-B, homoeostasis model assessment 2 estimates of β-cell function; HOMA2-IR, homoeostasis model assessment 2 estimates of insulin resistance.


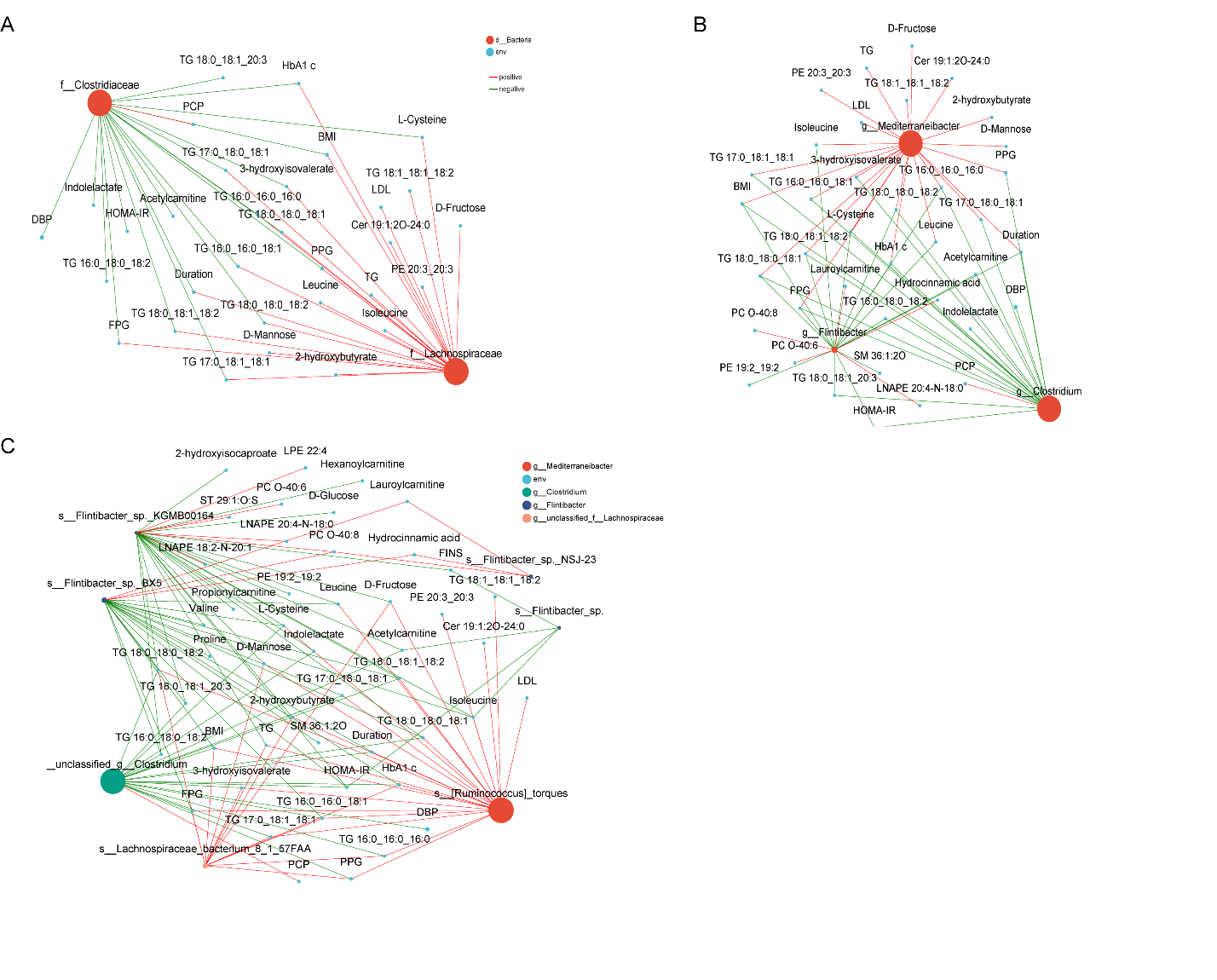


Supplementary Figure 6. Correlations among gut microbiota, host serum metabolites, and host clinical phenotypes in the context of UDM.

A. Gut microbiota in family level. B. Gut microbiota in genus level. C. Gut microbiota in species level. Red lines indicate positive correlations (P Value <0.05), and blue lines indicate negative correlations (P Value<0.05). PCP, 2-h postprandial C-peptide; PPG, 2-h postprandial glucose; FCP, fasting C-peptide.

Supplementary Table 1. The bacteria differentially abundance between patients with HC and UDM.

| Species | Mean relative abundance | | Lefse Analysis | |
| --- | --- | --- | --- | --- |
|  | HC-Mean(%) | UDM-Mean(%) | LDA score | Pvalue |
| f__Actinomycetaceae | 0.01098 | 0.07199 | 2.495975492 | 0.026780526 |
| f__Akkermansiaceae | 0.8751 | 0.05825 | 3.620595632 | 0.014843617 |
| f__Akkermansiaceae.g__Akkermansia | 0.8748 | 0.05824 | 3.620405303 | 0.014843617 |
| f__Akkermansiaceae.g__Akkermansia.s__Akkermansia_muciniphila | 0.7825 | 0.02638 | 3.586935029 | 0.014843617 |
| f__Akkermansiaceae.g__Akkermansia.s__Akkermansia_sp_ | 0.02583 | 0.001436 | 2.092848493 | 0.000350011 |
| f__Akkermansiaceae.g__Akkermansia.s__unclassified_g__Akkermansia | 0.02076 | 0.0006865 | 2.034087078 | 0.003924049 |
| f__Atopobiaceae.g__Olsenella.s__Olsenella_sp__AF21_51 | 0.0003568 | 0.02232 | 2.140508927 | 0.04897207 |
| f__Bacteroidaceae.g__Bacteroides.s__Bacteroides_faecis | 0.0915 | 0.04348 | 2.478548204 | 0.039733481 |
| f__Bacteroidaceae.g__Bacteroides.s__Bacteroides_plebeius_CAG_211 | 0.08962 | 0.0128 | 2.578343454 | 0.036784523 |
| f__Bacteroidaceae.g__Bacteroides.s__Bacteroides_sp__CAG_530 | 0.02684 | 0.00128 | 2.199597643 | 0.008639386 |
| f__Bacteroidaceae.g__Bacteroides.s__uncultured_Bacteroides_sp_ | 0.04653 | 0.02673 | 2.066249756 | 0.036784523 |
| f__Bacteroidaceae.g__Phocaeicola.s__Phocaeicola_plebeius | 3.15 | 0.4657 | 4.119282321 | 0.005913164 |
| f__Bacteroidaceae.g__unclassified_f__Bacteroidaceae.s__Bacteroidaceae_bacterium | 0.08106 | 0.04349 | 2.332170766 | 0.014843617 |
| f__Barnesiellaceae.g__Coprobacter.s__Coprobacter_fastidiosus | 0.1215 | 0.01372 | 2.711596215 | 0.017649281 |
| f__Bifidobacteriaceae | 0.4151 | 2.299 | 3.97645362 | 0.042880675 |
| f__Bifidobacteriaceae.g__Bifidobacterium | 0.4037 | 2.226 | 3.960402873 | 0.036784523 |
| f__Bifidobacteriaceae.g__Bifidobacterium.s__Bifidobacterium_longum | 0.0871 | 0.5327 | 3.346513077 | 0.016193118 |
| f__Bifidobacteriaceae.g__Gardnerella | 0.000539 | 0.02721 | 2.228763508 | 0.042827149 |
| f__Bifidobacteriaceae.g__Gardnerella.s__Gardnerella_vaginalis | 0.0004673 | 0.02 | 2.098776585 | 0.046179924 |
| f__Christensenellaceae | 0.06072 | 0.04937 | 2.184887276 | 0.026780526 |
| f__Christensenellaceae.g__unclassified_f__Christensenellaceae | 0.05685 | 0.04337 | 2.200325 | 0.013594216 |
| f__Christensenellaceae.g__unclassified_f__Christensenellaceae.s__Christensenellaceae_bacterium | 0.05685 | 0.04337 | 2.200325 | 0.013594216 |
| f__Clostridiaceae | 3.571 | 2.889 | 3.521361958 | 0.029031142 |
| f__Clostridiaceae.g__Clostridium | 2.969 | 2.041 | 3.636840366 | 0.001556917 |
| f__Clostridiaceae.g__Clostridium.s__Clostridium_sp__26_22 | 0.002667 | 0.03089 | 2.244130016 | 0.027295593 |
| f__Clostridiaceae.g__Clostridium.s__Clostridium_sp__28_17 | 0.002981 | 0.2245 | 3.124549689 | 0.022544917 |
| f__Clostridiaceae.g__Clostridium.s__Clostridium_sp__AF27_2AA | 0.02436 | 0.005831 | 2.004652571 | 6.71E-05 |
| f__Clostridiaceae.g__Clostridium.s__Clostridium_sp__AF37_5 | 0.02827 | 0.0004615 | 2.117743199 | 0.003596971 |
| f__Clostridiaceae.g__Clostridium.s__Clostridium_sp__AM22_11AC | 0.03001 | 0.009314 | 2.060548895 | 0.000797412 |
| f__Clostridiaceae.g__Clostridium.s__Clostridium_sp__AM33_3 | 0.04801 | 0.009943 | 2.308795694 | 2.96E-05 |
| f__Clostridiaceae.g__Clostridium.s__Clostridium_sp__CAG_417 | 0.02499 | 8.70E-05 | 2.067790891 | 0.017982083 |
| f__Clostridiaceae.g__Clostridium.s__Clostridium_sp__CAG_440 | 0.01596 | 0.05369 | 2.394481593 | 0.012408467 |
| f__Clostridiaceae.g__Clostridium.s__Clostridium_sp__CAG_470 | 0.000686 | 0.0447 | 2.408862498 | 0.047740312 |
| f__Clostridiaceae.g__Clostridium.s__Clostridium_sp__CAG_768 | 0.2065 | 0 | 3.02673001 | 0.036876781 |
| f__Clostridiaceae.g__Clostridium.s__Clostridium_sp__TM06_18 | 0.03107 | 0.009544 | 2.08359016191 | 0.000563485 |
| f__Clostridiaceae.g__Clostridium.s__unclassified_g__Clostridium | 0.8119 | 0.2483 | 3.450768859 | 1.46E-05 |
| f__Clostridiaceae.g__Clostridium.s__uncultured_Clostridium_sp_ | 0.197 | 0.1309 | 2.481098162 | 0.017649281 |
| f__Clostridiaceae.g__unclassified_f__Clostridiaceae | 0.3152 | 0.2016 | 2.751545 | 0.031442686 |
| f__Clostridiaceae.g__unclassified_f__Clostridiaceae.s__Clostridiaceae_bacterium_CLA_AA_H274 | 0.03669 | 0.003248 | 2.265618251 | 0.049809866 |
| f__Coriobacteriaceae | 0.04685 | 0.3407 | 3.164649304 | 0.016193118 |
| f__Coriobacteriaceae.g__Collinsella | 0.04462 | 0.3338 | 3.156910241 | 0.016193118 |
| f__Coriobacteriaceae.g__Collinsella.s__Collinsella_sp_ | 0.005041 | 0.0303 | 2.096059838 | 0.004865059 |
| f__Coriobacteriaceae.g__Collinsella.s__unclassified_g__Collinsella | 0.01096 | 0.1224 | 2.743179362 | 0.036784523 |
| f__Eggerthellaceae | 0.03754 | 0.3807 | 3.254035124 | 0.042880675 |
| f__Eggerthellaceae.g__Eggerthella | 0.01731 | 0.2246 | 3.050083926 | 0.001395895 |
| f__Eggerthellaceae.g__Eggerthella.s__Eggerthella_lenta | 0.006205 | 0.08913 | 2.650306905 | 0.001118941 |
| f__Eggerthellaceae.g__Eggerthella.s__unclassified_g__Eggerthella | 0.006279 | 0.1191 | 2.788297934 | 0.00264999 |
| f__Enterobacteriaceae | 1.46 | 4.6 | 4.175647155 | 0.000633225 |
| f__Enterobacteriaceae.g__Citrobacter | 0.2266 | 0.03153 | 3.025233076 | 0.022727818 |
| f__Enterobacteriaceae.g__Citrobacter.s__Citrobacter_freundii | 0.07079 | 0.006586 | 2.538472155 | 0.031442686 |
| f__Enterobacteriaceae.g__Citrobacter.s__Citrobacter_portucalensis | 0.036 | 0.003137 | 2.248238303 | 0.016186263 |
| f__Enterobacteriaceae.g__Enterobacter | 0.02529 | 0.05198 | 2.150041454 | 0.001250355 |
| f__Enterobacteriaceae.g__Escherichia | 0.5409 | 2.176 | 3.895984259 | 0.00360561 |
| f__Enterobacteriaceae.g__Escherichia.s__Escherichia_coli | 0.5294 | 2.111 | 3.882134576 | 0.00360561 |
| f__Enterobacteriaceae.g__Escherichia.s__unclassified_g__Escherichia | 0.008106 | 0.04805 | 2.264880399 | 0.005913164 |
| f__Enterobacteriaceae.g__Klebsiella | 0.2467 | 0.4372 | 3.064347563 | 0.003987959 |
| f__Enterobacteriaceae.g__Klebsiella.s__Klebsiella_pneumoniae | 0.1186 | 0.2353 | 2.778592809 | 0.002148167 |
| f__Enterobacteriaceae.g__Klebsiella.s__unclassified_g__Klebsiella | 0.1023 | 0.1738 | 2.719579893 | 0.042880675 |
| f__Enterobacteriaceae.g__Salmonella | 0.04683 | 0.09477 | 2.351121851 | 0.020909324 |
| f__Enterobacteriaceae.g__Salmonella.s__Salmonella_enterica | 0.04569 | 0.09202 | 2.33548304 | 0.024682201 |
| f__Enterobacteriaceae.g__Shigella | 0.0232 | 0.1237 | 2.691592124 | 0.005913164 |
| f__Enterobacteriaceae.g__Shigella.s__Shigella_flexneri | 0.008501 | 0.0414 | 2.206903147 | 0.005366047 |
| f__Enterobacteriaceae.g__Shigella.s__Shigella_sonnei | 0.008465 | 0.04957 | 2.299169752 | 0.005913164 |
| f__Enterobacteriaceae.g__unclassified_f__Enterobacteriaceae | 0.301 | 1.666 | 3.818646527 | 0.001931402 |
| f__Enterobacteriaceae.g__unclassified_f__Enterobacteriaceae.s__unclassified_f__Enterobacteriaceae | 0.3005 | 1.664 | 3.818194129 | 0.001931402 |
| f__Enterococcaceae.g__Enterococcus.s__Enterococcus_faecalis | 0.002971 | 0.2003 | 2.914900807 | 0.017649281 |
| f__Enterococcaceae.g__Enterococcus.s__Enterococcus_lactis | 0 | 0.03314 | 2.148325393 | 0.000310976 |
| f__Erysipelotrichaceae | 0.3472 | 0.8092 | 3.39139811 | 0.042880675 |
| f__Erysipelotrichaceae.g__Erysipelatoclostridium | 0.0972 | 0.477 | 3.282750744 | 0.016193118 |
| f__Erysipelotrichaceae.g__Erysipelatoclostridium.s___Clostridium__innocuum | 0.04916 | 0.1153 | 2.532860108 | 0.026780526 |
| f__Erysipelotrichaceae.g__Erysipelatoclostridium.s__unclassified_g__Erysipelatoclostridium | 0.02184 | 0.2218 | 3.002902013 | 0.00264999 |
| f__Eubacteriaceae.g__Eubacterium | 1.039 | 0.8321 | 3.132034888 | 0.049809866 |
| f__Eubacteriaceae.g__Eubacterium.s__Eubacterium_sp_ | 0.408 | 0.1922 | 3.037576042 | 0.016193118 |
| f__Eubacteriaceae.g__Eubacterium.s__Eubacterium_sp__CAG_38 | 0.03383 | 0.01201 | 2.019396772 | 0.00173489 |
| f__Eubacteriaceae.g__Eubacterium.s__Eubacterium_ventriosum | 0.1222 | 0.05598 | 2.543169562 | 0.026780526 |
| f__Lachnospiraceae.g__Acetatifactor | 0.06017 | 0.01594 | 2.372288605 | 0.01038538 |
| f__Lachnospiraceae.g__Acetatifactor.s__Acetatifactor_sp_ | 0.05494 | 0.01164 | 2.36630733 | 0.014843617 |
| f__Lachnospiraceae.g__Anaerotignum | 0.09239 | 0.04879 | 2.254322038 | 0.020909324 |
| f__Lachnospiraceae.g__Blautia.s__Blautia_sp__KLE_1732 | 0.008743 | 0.1415 | 2.784023906 | 0.016193118 |
| f__Lachnospiraceae.g__Blautia.s__Blautia_sp__SC05B48 | 0.00178 | 0.04759 | 2.34289018 | 0.016193118 |
| f__Lachnospiraceae.g__Blautia.s__uncultured_Blautia_sp_ | 0.01774 | 0.06719 | 2.399574126 | 0.022727818 |
| f__Lachnospiraceae.g__Butyrivibrio | 0.1075 | 0.009625 | 2.617455984 | 0.031442686 |
| f__Lachnospiraceae.g__Butyrivibrio.s__Butyrivibrio_crossotus | 0.0657 | 0.002058 | 2.431633373 | 0.005913164 |
| f__Lachnospiraceae.g__Enterocloster | 0.3622 | 0.6525 | 3.167729129 | 0.049809866 |
| f__Lachnospiraceae.g__Enterocloster.s__Enterocloster_bolteae | 0.09873 | 0.2364 | 2.779252108 | 0.039733481 |
| f__Lachnospiraceae.g__Enterocloster.s__unclassified_g__Enterocloster | 0.05643 | 0.1643 | 2.676367212 | 0.042880675 |
| f__Lachnospiraceae.g__Lachnospira | 1.406 | 0.2897 | 3.751683771 | 0.000500954 |
| f__Lachnospiraceae.g__Lachnospira.s__Lachnospira_eligens | 0.7 | 0.07043 | 3.516565059 | 0.005366047 |
| f__Lachnospiraceae.g__Lachnospira.s__Lachnospira_pectinoschiza | 0.3862 | 0.1513 | 3.083345639 | 0.01038538 |
| f__Lachnospiraceae.g__Lachnospira.s__Lachnospira_sp_ | 0.2481 | 0.05796 | 2.960416543 | 0.000633225 |
| f__Lachnospiraceae.g__Lachnospira.s__Lachnospira_sp__NSJ_43 | 0.06336 | 0.004502 | 2.433469971 | 0.001556917 |
| f__Lachnospiraceae.g__Mediterraneibacter | 0.4479 | 1.798 | 3.843837754 | 0.000710927 |
| f__Lachnospiraceae.g__Mediterraneibacter.s___Ruminococcus__gnavus | 0.1472 | 0.4683 | 3.167258692 | 0.007868934 |
| f__Lachnospiraceae.g__Mediterraneibacter.s___Ruminococcus__torques | 0.1489 | 0.9173 | 3.621634624 | 5.13E-05 |
| f__Lachnospiraceae.g__Mediterraneibacter.s__Mediterraneibacter_glycyrrhizinilyticus | 0.004641 | 0.05812 | 2.390350088 | 0.00651006 |
| f__Lachnospiraceae.g__Mediterraneibacter.s__unclassified_g__Mediterraneibacter | 0.009897 | 0.06448 | 2.448582998 | 0.013594216 |
| f__Lachnospiraceae.g__Simiaoa | 0.09959 | 0.01787 | 2.669646206 | 0.014843617 |
| f__Lachnospiraceae.g__Simiaoa.s__Simiaoa_sunii | 0.09959 | 0.01787 | 2.669646206 | 0.014843617 |
| f__Lachnospiraceae.g__unclassified_f__Lachnospiraceae.s__Lachnospiraceae_bacterium_8_1_57FAA | 0.00308 | 0.0245 | 2.068886179 | 5.13E-05 |
| f__Lachnospiraceae.g__Waltera | 0.03733 | 0.007823 | 2.191240143 | 0.00360561 |
| f__Lachnospiraceae.g__Waltera.s__Waltera_intestinalis | 0.02129 | 0.0032 | 2.015688691 | 0.010380448 |
| f__Lactobacillaceae.g__Limosilactobacillus | 0.004193 | 0.09651 | 2.7056031 | 0.046236071 |
| f__Morganellaceae | 0.01776 | 0.5308 | 3.270967017 | 0.026780526 |
| f__Morganellaceae.g__Morganella | 0.00177 | 0.4986 | 3.250661926 | 0.002023574 |
| f__Morganellaceae.g__Morganella.s__Morganella_morganii | 0.0005713 | 0.206 | 2.867308988 | 0.005320122 |
| f__Morganellaceae.g__Morganella.s__unclassified_g__Morganella | 0.001189 | 0.2906 | 3.016149898 | 0.0018183 |
| f__Muribaculaceae | 0.2085 | 0.032 | 2.954023796 | 0.00360561 |
| f__Muribaculaceae.g__unclassified_f__Muribaculaceae | 0.1513 | 0.01098 | 2.858089195 | 0.000797412 |
| f__Muribaculaceae.g__unclassified_f__Muribaculaceae.s__Muribaculaceae_bacterium | 0.1188 | 0.008415 | 2.756182399 | 0.00440677 |
| f__Oscillospiraceae.g__Dysosmobacter.s__Dysosmobacter_sp_ | 0.04131 | 0.01078 | 2.191464123 | 0.00173489 |
| f__Oscillospiraceae.g__Dysosmobacter.s__Dysosmobacter_sp__BX15 | 0.08414 | 0.02718 | 2.47178266 | 0.007868934 |
| f__Oscillospiraceae.g__Faecalibacterium | 5.087 | 1.848 | 4.199361124 | 0.00264999 |
| f__Oscillospiraceae.g__Faecalibacterium.s__Faecalibacterium_duncaniae | 0.05609 | 0.02159 | 2.208495695 | 0.001931402 |
| f__Oscillospiraceae.g__Faecalibacterium.s__Faecalibacterium_prausnitzii | 2.404 | 0.9144 | 3.857412882 | 0.005366047 |
| f__Oscillospiraceae.g__Faecalibacterium.s__Faecalibacterium_sp_ | 0.7329 | 0.2549 | 3.386821187 | 0.002148167 |
| f__Oscillospiraceae.g__Faecalibacterium.s__Faecalibacterium_sp__CAG_74 | 0.02374 | 0.0001461 | 2.10042769 | 0.001845454 |
| f__Oscillospiraceae.g__Faecalibacterium.s__Faecalibacterium_sp__CLA_AA_H233 | 0.00716 | 0.002093 | 2.194119116 | 0.001931402 |
| f__Oscillospiraceae.g__Faecalibacterium.s__Faecalibacterium_sp__I3389 | 0.02724 | 0.004555 | 2.033321232 | 0.00360561 |
| f__Oscillospiraceae.g__Faecalibacterium.s__Faecalibacterium_sp__Marseille_Q3530 | 0.02579 | 0.004451 | 2.016316908 | 0.003256897 |
| f__Oscillospiraceae.g__Faecalibacterium.s__Faecalibacterium_sp__OF04_11AC | 0.04379 | 0.007598 | 2.272524316 | 0.001250355 |
| f__Oscillospiraceae.g__Faecalibacterium.s__unclassified_g__Faecalibacterium | 1.188 | 0.4223 | 3.571077903 | 0.001395895 |
| f__Oscillospiraceae.g__Faecalibacterium.s__uncultured_Faecalibacterium_sp_ | 0.04116 | 0.01577 | 2.08377137 | 0.000797412 |
| f__Oscillospiraceae.g__Lawsonibacter | 0.05827 | 0.02261 | 2.234752136 | 0.00440677 |
| f__Oscillospiraceae.g__Oscillibacter | 0.7147 | 0.389 | 3.172544098 | 0.017649281 |
| f__Oscillospiraceae.g__Oscillibacter.s__Oscillibacter_sp_ | 0.3361 | 0.2124 | 2.768263358 | 0.022727818 |
| f__Oscillospiraceae.g__Oscillibacter.s__Oscillibacter_sp__ER4 | 0.0649 | 0.02807 | 2.280051967 | 0.013594216 |
| f__Oscillospiraceae.g__Oscillibacter.s__Oscillibacter_sp__MCC667 | 0.08817 | 0.02414 | 2.511843072 | 0.019218964 |
| f__Oscillospiraceae.g__Oscillibacter.s__Oscillibacter_sp__MSJ_31 | 0.02977 | 0.004835 | 2.093419236 | 0.01038538 |
| f__Oscillospiraceae.g__Oscillibacter.s__Oscillibacter_valericigenes | 0.07328 | 0.02555 | 2.378466655 | 0.001118941 |
| f__Oscillospiraceae.g__Pseudoflavonifractor | 0.07873 | 0.04069 | 2.238827421 | 0.008639386 |
| f__Oscillospiraceae.g__Ruminococcus.s__Ruminococcus_sp__210702_SL_1_03 | 0.02682 | 0.001236 | 2.062465023 | 0.041281657 |
| f__Oscillospiraceae.g__Ruminococcus.s__Ruminococcus_sp__OF02_6 | 0.002592 | 0.04219 | 2.275854776 | 0.046236071 |
| f__Oscillospiraceae.g__Ruminococcus.s__Ruminococcus_sp__SR1_5 | 0.001946 | 0.03394 | 2.166314014 | 0.046236071 |
| f__Oscillospiraceae.g__Subdoligranulum.s__Subdoligranulum_sp__APC924_74 | 0.1705 | 0.03881 | 2.804548274 | 0.029031142 |
| f__Oscillospiraceae.g__unclassified_f__Oscillospiraceae | 1.612 | 1.296 | 3.357694099 | 0.031442686 |
| f__Oscillospiraceae.g__unclassified_f__Oscillospiraceae.s__Ruminococcaceae_bacterium_TF06_43 | 0.03638 | 0.01596 | 2.01737828 | 0.031442686 |
| f__Pasteurellaceae.g__Haemophilus.s__Haemophilus_parainfluenzae | 0.03962 | 0.02065 | 2.0989356 | 0.019218964 |
| f__Porphyromonadaceae | 0.09789 | 0.08625 | 2.257751302 | 0.031442686 |
| f__Porphyromonadaceae.g__unclassified_f__Porphyromonadaceae | 0.04536 | 0.007624 | 2.369196859 | 0.013594216 |
| f__Porphyromonadaceae.g__unclassified_f__Porphyromonadaceae.s__Porphyromonadaceae_bacterium | 0.04514 | 0.007545 | 2.367686211 | 0.01038538 |
| f__Prevotellaceae | 16.26 | 1.128 | 4.914789501 | 0.022727818 |
| f__Prevotellaceae.g__Prevotella | 15.6 | 0.9772 | 4.896709214 | 0.014843617 |
| f__Prevotellaceae.g__Prevotella.s__Prevotella_hominis | 0.172 | 0.00625 | 3.023763397 | 0.005845878 |
| f__Prevotellaceae.g__Prevotella.s__Prevotella_marseillensis | 0.09667 | 0.001064 | 2.659674795 | 0.042880675 |
| f__Prevotellaceae.g__Prevotella.s__Prevotella_pectinovora | 0.1349 | 0.0004447 | 2.879300239 | 0.000633225 |
| f__Prevotellaceae.g__Prevotella.s__Prevotella_rara | 0.1052 | 0.0005072 | 2.698602792 | 0.016193118 |
| f__Prevotellaceae.g__Prevotella.s__Prevotella_sp_ | 1.338 | 0.0687 | 3.85818128 | 0.00173489 |
| f__Prevotellaceae.g__Prevotella.s__Prevotella_sp__CAG_255 | 0.08787 | 0.0009204 | 2.618787178 | 0.016193118 |
| f__Prevotellaceae.g__Prevotella.s__Prevotella_sp__CAG_520 | 0.04301 | 0.0006875 | 2.390455365 | 0.019626802 |
| f__Prevotellaceae.g__Prevotella.s__Prevotella_sp__CAG_891 | 0.03224 | 0.002287 | 2.245060028 | 0.046236071 |
| f__Prevotellaceae.g__Prevotella.s__Prevotella_stercorea | 0.4417 | 0.00789 | 3.361641562 | 0.022727818 |
| f__Prevotellaceae.g__Prevotella.s__Prevotella_stercorea_CAG_629 | 0.0256 | 0.0002676 | 2.076540046 | 0.0174792 |
| f__Prevotellaceae.g__Prevotella.s__unclassified_g__Prevotella | 1.614 | 0.1375 | 3.910154967 | 0.03402408 |
| f__Prevotellaceae.g__Prevotellamassilia | 0.4159 | 0.0006089 | 3.429872742 | 0.000309996 |
| f__Prevotellaceae.g__Prevotellamassilia.s__Prevotellamassilia_timonensis | 0.3529 | 0.0005259 | 3.358356834 | 0.000444941 |
| f__Pseudomonadaceae | 0.01946 | 0.06399 | 2.283631955 | 0.014843617 |
| f__Pseudomonadaceae.g__Pseudomonas | 0.01938 | 0.06367 | 2.280627634 | 0.020909324 |
| f__Pseudomonadaceae.g__Pseudomonas.s__Pseudomonas_aeruginosa | 0.008252 | 0.04941 | 2.25541113 | 0.019218964 |
| f__Streptococcaceae.g__Streptococcus.s__unclassified_g__Streptococcus | 0.01917 | 0.1798 | 2.878196796 | 0.00651006 |
| f__Streptomycetaceae | 0.008716 | 0.04098 | 2.152456416 | 0.042880675 |
| f__Streptomycetaceae.g__Streptomyces | 0.008716 | 0.04095 | 2.152031917 | 0.042880675 |
| f__Tannerellaceae.g__Tannerella | 0.08932 | 0.0202 | 2.52402757 | 0.005913164 |
| f__Tannerellaceae.g__Tannerella.s__Tannerella_sp__AF04_6 | 0.0305 | 0.002885 | 2.126312819 | 0.012946781 |
| f__Turicibacteraceae | 0.006284 | 0.03617 | 2.106257717 | 0.042880675 |
| f__Turicibacteraceae.g__Turicibacter | 0.006284 | 0.03617 | 2.106257717 | 0.042880675 |
| f__unclassified_c__Bacilli.g__unclassified_c__Bacilli.s__Bacilli_bacterium | 0.048 | 0.02397 | 2.068095675 | 0.008639386 |
| f__unclassified_c__Gammaproteobacteria | 0.0198 | 0.06853 | 2.363412767 | 0.002387033 |
| f__unclassified_c__Gammaproteobacteria.g__unclassified_c__Gammaproteobacteria | 0.01954 | 0.06849 | 2.365226912 | 0.001931402 |
| f__unclassified_c__Gammaproteobacteria.g__unclassified_c__Gammaproteobacteria.s__unclassified_c__Gammaproteobacteria | 0.09169 | 0.0496 | 2.414475694 | 0.001250355 |
| f__unclassified_d__Bacteria.g__unclassified_d__Bacteria.s__bacterium | 0.1503 | 0.03035 | 2.358284458 | 0.01038538 |
| f__unclassified_o__Bacteroidales.g__unclassified_o__Bacteroidales.s__Bacteroidales_bacterium | 0.1503 | 0.03035 | 2.830364317 | 0.00264999 |
| f__unclassified_o__Enterobacterales | 0.01902 | 0.1025 | 2.559077816 | 0.001931402 |
| f__unclassified_o__Enterobacterales.g__unclassified_o__Enterobacterales | 0.01902 | 0.1025 | 2.559077816 | 0.001931402 |
| f__unclassified_o__Enterobacterales.g__unclassified_o__Enterobacterales.s__unclassified_o__Enterobacterales | 0.01902 | 0.1025 | 2.559050035 | 0.001931402 |
| f__unclassified_o__Eubacteriales.g__Flintibacter | 0.04673 | 0.006605 | 2.310458764 | 2.24E-05 |
| f__unclassified_o__Eubacteriales.g__Flintibacter.s__Flintibacter_sp__BX5 | 0.03632 | 0.004024 | 2.220311446 | 0.000214146 |
| f__unclassified_o__Eubacteriales.g__unclassified_o__Eubacteriales.s__Clostridiales_bacterium | 1.287 | 0.8363 | 3.266115287 | 0.036784523 |
| f__unclassified_p__Firmicutes.g__unclassified_p__Firmicutes.s__Firmicutes_bacterium_AF22_6AC | 0.03575 | 0.006368 | 2.220822542 | 0.00264999 |
| f__unclassified_p__Firmicutes.g__unclassified_p__Firmicutes.s__Firmicutes_bacterium_AM59_13 | 0.05519 | 0.009438 | 2.419776402 | 0.00440677 |
| f__unclassified_p__Firmicutes.g__unclassified_p__Firmicutes.s__Firmicutes_bacterium_CAG_341 | 0.07083 | 0.06775 | 2.34388526 | 0.02082703 |
| f__unclassified_p__Firmicutes.g__unclassified_p__Firmicutes.s__Firmicutes_bacterium_CAG_65 | 0.02296 | 0.003764 | 2.038880603 | 0.00651006 |
| f__unclassified_p__Firmicutes.g__unclassified_p__Firmicutes.s__Firmicutes_bacterium_OM08_11AC | 0.04662 | 0.008519 | 2.335694146 | 0.00264999 |
| f__Veillonellaceae.g__Dialister | 1.113 | 0.0708 | 3.761358376 | 0.005366047 |
| f__Veillonellaceae.g__Dialister.s__Dialister_invisus | 0.5904 | 0.01999 | 3.499135129 | 0.039733481 |
| f__Veillonellaceae.g__Dialister.s__Dialister_sp_ | 0.2395 | 0.01777 | 3.094387504 | 0.00651006 |
| f__Veillonellaceae.g__Dialister.s__Dialister_succinatiphilus | 0.09686 | 0.001546 | 2.630571128 | 0.038110608 |
| f__Veillonellaceae.g__Dialister.s__unclassified_g__Dialister | 0.07268 | 0.01207 | 2.577969108 | 0.043169769 |
| f__Vibrionaceae | 0.07912 | 0.01913 | 2.505939402 | 0.003987959 |
| f__Vibrionaceae.g__Vibrio | 0.07891 | 0.01874 | 2.507198177 | 0.002387033 |
| f__Vibrionaceae.g__Vibrio.s__Vibrio_parahaemolyticus | 0.07217 | 0.01527 | 2.480153961 | 0.000710927 |

Supplementary Table 2. The bacteria differentially abundance between patients with adult-onset T1DM and UDM.

| Species | Mean relative abundance | | Lefse Analysis | |
| --- | --- | --- | --- | --- |
|  | T1DM-Mean(%) | UDM-Mean(%) | LDA score | Pvalue |
| f__Bacteroidaceae | 16.7 | 35.59 | 4.896489111 | 0.00651006 |
| f__Bacteroidaceae.g__Bacteroides | 8.995 | 21.53 | 4.736162751 | 0.004865059 |
| f__Bacteroidaceae.g__Bacteroides.s__Bacteroides_cellulosilyticus | 0.07096 | 0.1976 | 2.830180801 | 0.007868934 |
| f__Bacteroidaceae.g__Bacteroides.s__Bacteroides_clarus | 0.0422 | 0.1501 | 2.749166797 | 0.029031142 |
| f__Bacteroidaceae.g__Bacteroides.s__Bacteroides_clarus_CAG_160 | 0.009455 | 0.03142 | 2.053689013 | 0.03402408 |
| f__Bacteroidaceae.g__Bacteroides.s__Bacteroides_fluxus | 0.007162 | 0.04759 | 2.196569095 | 0.008639386 |
| f__Bacteroidaceae.g__Bacteroides.s__Bacteroides_fragilis | 0.7681 | 3.478 | 3.928889091 | 0.022727818 |
| f__Bacteroidaceae.g__Bacteroides.s__Bacteroides_salyersiae | 0.02402 | 0.05934 | 2.173724088 | 0.008639386 |
| f__Bacteroidaceae.g__Bacteroides.s__Bacteroides_sp__3_1_33FAA | 0.02707 | 0.063 | 2.204240776 | 0.019218964 |
| f__Bacteroidaceae.g__Bacteroides.s__Bacteroides_sp__4_1_36 | 0.01412 | 0.03179 | 2.001848569 | 0.03402408 |
| f__Bacteroidaceae.g__Bacteroides.s__Bacteroides_sp__4_3_47FAA | 0.06101 | 0.1395 | 2.489058652 | 0.039733481 |
| f__Bacteroidaceae.g__Bacteroides.s__Bacteroides_sp__9_1_42FAA | 0.03153 | 0.06555 | 2.180514043 | 0.016193118 |
| f__Bacteroidaceae.g__Bacteroides.s__Bacteroides_sp__NSJ_48 | 0.03342 | 0.07306 | 2.328760156 | 0.049809866 |
| f__Bacteroidaceae.g__Bacteroides.s__Bacteroides_stercoris | 1.294 | 4.895 | 4.268740827 | 0.012438646 |
| f__Bacteroidaceae.g__Bacteroides.s__Bacteroides_stercoris_CAG_120 | 0.0252 | 0.1023 | 2.602587165 | 0.009476577 |
| f__Bacteroidaceae.g__Bacteroides.s__Bacteroides_xylanisolvens | 0.2216 | 0.6224 | 3.285103047 | 0.049809866 |
| f__Bacteroidaceae.g__Bacteroides.s__unclassified_g__Bacteroides | 2.366 | 5.756 | 4.1647213 | 0.008639386 |
| f__Bacteroidaceae.g__Phocaeicola.s__Phocaeicola_dorei | 1.241 | 2.998 | 3.873662621 | 0.031442686 |
| f__Bacteroidaceae.g__Phocaeicola.s__Phocaeicola_vulgatus | 1.991 | 4.317 | 3.952115951 | 0.042880675 |
| f__Bacteroidaceae.g__Phocaeicola.s__unclassified_g__Phocaeicola | 1.123 | 2.499 | 3.722763302 | 0.039733481 |
| f__Bacteroidaceae.g__unclassified_f__Bacteroidaceae | 1.108 | 2.263 | 3.670684381 | 0.019218964 |
| f__Bacteroidaceae.g__unclassified_f__Bacteroidaceae.s__unclassified_f__Bacteroidaceae | 1.082 | 2.22 | 3.663455142 | 0.020909324 |
| f__Chromatiaceae.g__Candidatus_Nitrosoglobus | 0 | 0.000009325 | 2.036634643 | 0.036876781 |
| f__Chromatiaceae.g__Candidatus_Nitrosoglobus.s__Candidatus_Nitrosoglobus_terrae | 0 | 0.000009325 | 2.076441322 | 0.036876781 |
| f__Clostridiaceae.g__Clostridium.s__Clostridium_celatum | 0.05011 | 0.009554 | 2.159402761 | 0.02082703 |
| f__Comamonadaceae.g__Delftia.s__Delftia_sp__K82 | 0 | 0.0000151 | 2.107539276 | 0.036876781 |
| f__Comamonadaceae.g__Delftia.s__Delftia_tsuruhatensis | 0 | 0.00001726 | 2.021985445 | 3.69E-02 |
| f__Coprobacillaceae.g__Catenibacterium | 0.01695 | 0.05615 | 2.260509332 | 0.001395895 |
| f__Coprobacillaceae.g__Catenibacterium.s__Catenibacterium_mitsuokai | 0.01586 | 0.04551 | 2.147646389 | 0.001395895 |
| f__Erysipelotrichaceae.g__Erysipelatoclostridium.s__unclassified_g__Erysipelatoclostridium | 0.03314 | 0.2218 | 2.831636286 | 4.29E-02 |
| f__Eubacteriaceae.g__Eubacterium.s__Eubacterium_eligens_CAG_72 | 0.01884 | 4.34E-04 | 2.033734329 | 0.011225368 |
| f__Fusobacteriaceae.g__Fusobacterium.s__Fusobacterium_ulcerans | 0.07863 | 0.05367 | 2.356853338 | 0.017620106 |
| f__Lachnospiraceae.g__Enterocloster.s__Enterocloster_sp_ | 0.01667 | 0.08248 | 2.433693447 | 0.026780526 |
| f__Lachnospiraceae.g__Enterocloster.s__unclassified_g__Enterocloster | 0.05948 | 0.1643 | 2.538443244 | 0.042880675 |
| f__Lachnospiraceae.g__Mediterraneibacter | 1.042 | 1.798 | 3.570177338 | 0.042880675 |
| f__Lachnospiraceae.g__Mediterraneibacter.s___Ruminococcus__torques | 0.5077 | 0.9173 | 3.364221527 | 1.14E-02 |
| f__Lactobacillaceae.g__Lacticaseibacillus | 0.6207 | 0.009772 | 3.517664745 | 0.003987959 |
| f__Lactobacillaceae.g__Lacticaseibacillus.s__Lacticaseibacillus_paracasei | 0.1966 | 0.002957 | 3.025794947 | 0.016193118 |
| f__Lactobacillaceae.g__Lacticaseibacillus.s__Lacticaseibacillus_rhamnosus | 0.03225 | 0.00633 | 2.015240548 | 0.009476577 |
| f__Microbacteriaceae.g__Microbacterium.s__Microbacterium_sp__AISO3 | 0.000004721 | 0 | 2.205081527 | 0.036876781 |
| f__Odoribacteraceae | 0.3261 | 0.6554 | 3.236982264 | 0.042880675 |
| f__Odoribacteraceae.g__Butyricimonas | 0.1004 | 0.2031 | 2.760801433 | 0.046236071 |
| f__Odoribacteraceae.g__Butyricimonas.s__Butyricimonas_virosa | 0.04034 | 0.07294 | 2.27434633 | 0.039733481 |
| f__Oscillospiraceae.g__Faecalibacterium | 4.968 | 1.848 | 4.21346266 | 0.020909324 |
| f__Oscillospiraceae.g__Faecalibacterium.s__Faecalibacterium_duncaniae | 0.06322 | 0.02159 | 2.351571006 | 0.014843617 |
| f__Oscillospiraceae.g__Faecalibacterium.s__Faecalibacterium_prausnitzii | 2.369 | 0.9144 | 3.895091619 | 0.046236071 |
| f__Oscillospiraceae.g__Faecalibacterium.s__Faecalibacterium_sp_ | 0.618 | 0.2549 | 3.266139517 | 0.042880675 |
| f__Oscillospiraceae.g__Faecalibacterium.s__Faecalibacterium_sp__AM43_5AT | 0.03948 | 0.009687 | 2.128759118 | 0.013594216 |
| f__Oscillospiraceae.g__Faecalibacterium.s__unclassified_g__Faecalibacterium | 1.257 | 0.4223 | 3.630756414 | 0.016193118 |
| f__Oscillospiraceae.g__Faecalibacterium.s__uncultured_Faecalibacterium_sp_ | 0.0563 | 0.01577 | 2.263336904 | 0.012438646 |
| f__Oscillospiraceae.g__Jilunia | 0.002053 | 0.03433 | 2.209084432 | 0.001930101 |
| f__Oscillospiraceae.g__Jilunia.s__Jilunia_laotingensis | 0.002053 | 0.03433 | 2.209084432 | 0.001930101 |
| f__Oscillospiraceae.g__Subdoligranulum.s__Subdoligranulum_sp_ | 0.2691 | 0.07701 | 2.943943051 | 0.031442686 |
| f__Oscillospiraceae.g__Subdoligranulum.s__Subdoligranulum_sp__APC924_74 | 0.1347 | 0.03881 | 2.664677973 | 0.049809866 |
| f__Paenibacillaceae.g__Paenibacillus.s__Paenibacillus_sp__BC26 | 0.000005484 | 0 | 2.116986603 | 0.036876781 |
| f__Peptostreptococcaceae | 0.4334 | 0.2243 | 3.014076284 | 0.036784523 |
| f__Peptostreptococcaceae.g__Peptostreptococcus.s__Peptostreptococcus_anaerobius_CAG_621 | 0 | 0.0002734 | 2.578426618 | 0.036876781 |
| f__Peptostreptococcaceae.g__Romboutsia | 0.1222 | 0.03405 | 2.648009657 | 0.019218964 |
| f__Peptostreptococcaceae.g__Romboutsia.s__Romboutsia_timonensis | 0.09331 | 0.02607 | 2.530131575 | 0.022727818 |
| f__Peptostreptococcaceae.g__unclassified_f__Peptostreptococcaceae.s__Peptostreptococcaceae_bacterium | 0.0619 | 0.01862 | 2.341219144 | 0.024682201 |
| f__Porphyromonadaceae | 0.03956 | 0.08625 | 2.380028058 | 0.029031142 |
| f__Porphyromonadaceae.g__Porphyromonas | 0.02188 | 0.06875 | 2.359882414 | 0.03402408 |
| f__Prevotellaceae.g__Prevotella.s__Prevotella_bivia | 0.02247 | 0.05686 | 2.267829117 | 0.049809866 |
| f__Prevotellaceae.g__Prevotella.s__Prevotella_saccharolytica | 0 | 0.0006809 | 2.443508371 | 0.036876781 |
| f__Streptococcaceae.g__Lactococcus.s__Lactococcus_lactis | 0.03659 | 0.00229 | 2.267738347 | 0.042880675 |
| f__Streptococcaceae.g__Streptococcus.s__Streptococcus_sp__959 | 0.000004091 | 0 | 2.024019197 | 0.036876781 |
| f__Sutterellaceae.g__Mesosutterella | 0.0002311 | 0.02839 | 2.138727376 | 0.004107782 |
| f__Sutterellaceae.g__Mesosutterella.s__Mesosutterella_multiformis | 0.00007622 | 0.02577 | 2.106103546 | 0.018297227 |
| f__Sutterellaceae.g__Parasutterella.s__Parasutterella_secunda | 0.000214 | 0.01856 | 2.003795381 | 0.042132714 |
| f__Tannerellaceae.g__Parabacteroides.s__Parabacteroides_goldsteinii | 0.0246 | 0.5119 | 3.392902229 | 0.031442686 |
| f__unclassified_c__Bacilli.g__unclassified_c__Bacilli.s__Bacilli_bacterium | 0.02791 | 0.02397 | 2.036161188 | 0.008639386 |
| f__unclassified_c__Betaproteobacteria.g__Candidatus_Accumulibacter.s__Candidatus_Accumulibacter_phosphatis | 0 | 0.0002168 | 2.112652925 | 0.036876781 |
| f__unclassified_o__Bacteroidales | 1.848 | 2.946 | 3.67084059 | 0.042880675 |
| f__unclassified_o__Bacteroidales.g__unclassified_o__Bacteroidales | 1.848 | 2.945 | 3.670671554 | 0.042880675 |
| f__unclassified_o__Bacteroidales.g__unclassified_o__Bacteroidales.s__unclassified_o__Bacteroidales | 1.805 | 2.897 | 3.667098942 | 0.049809866 |
| f__unclassified_o__Burkholderiales.g__Paucibacter | 0 | 0.00001912 | 2.232526692 | 0.036876781 |
| f__unclassified_o__Eubacteriales.g__Gemmiger | 1.29 | 0.2061 | 3.690473227 | 0.031442686 |
| f__unclassified_o__Eubacteriales.g__Gemmiger.s__Gemmiger_sp_ | 0.2844 | 0.05085 | 3.032247824 | 0.008639386 |
| f__unclassified_p__Bacteroidota | 1.848 | 2.946 | 2.276564442 | 0.042880675 |
| f__unclassified_p__Bacteroidota.g__unclassified_p__Bacteroidota | 0.04836 | 0.08602 | 2.276564442 | 0.042880675 |
| f__unclassified_p__Bacteroidota.g__unclassified_p__Bacteroidota.s__unclassified_p__Bacteroidota | 0.035 | 0.06126 | 2.072686297 | 0.022727818 |

Supplementary Table 3. The bacteria differentially abundance between patients with adult-onset T2DM and UDM.

| Species | Mean relative abundance | | Lefse Analysis | |
| --- | --- | --- | --- | --- |
|  | T2DM-Mean(%) | UDM-Mean(%) | LDA score | Pvalue |
| f__Actinomycetaceae.g__Actinomyces.s__Actinomyces_bouchesdurhonensis | 0.03105 | 0.001459 | 2.210732724 | 0.027694959 |
| f__Actinomycetaceae.g__Actinomyces.s__Actinomyces_sp_ | 0.05707 | 0.003852 | 2.460691 | 0.04215345 |
| f__Actinomycetaceae.g__Actinomyces.s__Actinomyces_sp__ICM47 | 0.04084 | 0.002726 | 2.318349956 | 0.038048172 |
| f__Bacteroidaceae.g__Bacteroides | 10.95 | 21.53 | 4.753582434 | 0.027712279 |
| f__Bacteroidaceae.g__Bacteroides.s__Bacteroides_coprocola_CAG_162 | 0.03836 | 0.006063 | 2.228719886 | 0.015821715 |
| f__Bacteroidaceae.g__Bacteroides.s__Bacteroides_eggerthii | 0.0537 | 0.1693 | 2.783160364 | 0.027712279 |
| f__Bacteroidaceae.g__Bacteroides.s__Bacteroides_fluxus | 0.00688 | 0.04759 | 2.228621044 | 0.01250128 |
| f__Bacteroidaceae.g__Bacteroides.s__Bacteroides_sp__3_1_33FAA | 0.03418 | 0.063 | 2.229117355 | 0.030849999 |
| f__Bacteroidaceae.g__Bacteroides.s__Bacteroides_sp__NSJ_48 | 0.01699 | 0.07306 | 2.524608772 | 0.034287968 |
| f__Bacteroidaceae.g__Bacteroides.s__Bacteroides_stercoris | 1.091 | 4.895 | 4.353880761 | 0.019894287 |
| f__Bacteroidaceae.g__Bacteroides.s__Bacteroides_stercoris_CAG_120 | 0.02285 | 0.1023 | 2.672909999 | 0.022254125 |
| f__Bacteroidaceae.g__Bacteroides.s__unclassified_g__Bacteroides | 2.869 | 5.756 | 4.175882528 | 0.046627448 |
| f__Barnesiellaceae.g__Coprobacter.s__Coprobacter_secundus | 0.01209 | 0.02904 | 2.026891775 | 0.019894287 |
| f__Burkholderiaceae.g__Caballeronia.s__Caballeronia_hypogeia | 0.000003435 | 0 | 2.099133489 | 0.028172613 |
| f__Candidatus_Nanosynbacteraceae | 0.02747 | 0.003087 | 2.133077514 | 0.017705285 |
| f__Candidatus_Nanosynbacteraceae.g__Candidatus_Nanosynbacter | 0.02747 | 0.003087 | 2.133077514 | 0.017705285 |
| f__Carnobacteriaceae | 0.02403 | 0.002771 | 2.067861755 | 0.038048172 |
| f__Clostridiaceae.g__Clostridium.s__Clostridium_tyrobutyricum | 0.02018 | 0.0001805 | 2.038454729 | 0.028618172 |
| f__Coprobacillaceae.g__Catenibacterium | 0.02237 | 0.05615 | 2.319983616 | 0.04215345 |
| f__Coprobacillaceae.g__Catenibacterium.s__Catenibacterium_mitsuokai | 0.02134 | 0.04551 | 2.177472405 | 0.034287968 |
| f__Erysipelotrichaceae.g__Solobacterium.s__Solobacterium_sp_ | 0.04468 | 0.003981 | 2.336242097 | 0.04215345 |
| f__Eubacteriaceae.g__Eubacterium.s__Eubacterium_ramulus | 0.05053 | 0.03061 | 2.026099006 | 0.046627448 |
| f__Lachnospiraceae.g__Enterocloster.s__Enterocloster_hominis | 0.1339 | 0.01078 | 2.807055108 | 0.007653106 |
| f__Lachnospiraceae.g__Roseburia.s__Roseburia_sp__CAG_471 | 0.02182 | 0.001776 | 2.045085008 | 0.037962539 |
| f__Lactobacillaceae.g__Weissella.s__Weissella_viridescens | 0.02418 | 0.001341 | 2.131992588 | 0.046502665 |
| f__Micrococcaceae.g__Rothia.s__Rothia_mucilaginosa | 0.04198 | 0.003153 | 2.33003175 | 0.046627448 |
| f__Odoribacteraceae.g__Butyricimonas | 0.07973 | 0.2031 | 2.869099781 | 4.66E-02 |
| f__Prevotellaceae.g__Prevotella.s__Prevotella_sp__CAG_520 | 0.02816 | 0.0006875 | 2.161824281 | 0.012828236 |
| f__Prevotellaceae.g__Prevotella.s__Prevotella_sp__Marseille_P4119 | 0.5028 | 0.0004153 | 3.39577332 | 0.048164057 |
| f__Prevotellaceae.g__Prevotella.s__Prevotella_stercorea_CAG_629 | 0.0571 | 0.0002676 | 2.471466508 | 1.48E-02 |
| f__Selenomonadaceae.g__Megamonas.s__Megamonas_funiformis | 0.8693 | 9.48E-02 | 3.63326914 | 0.027712279 |
| f__Selenomonadaceae.g__Megamonas.s__Megamonas_rupellensis | 0.2878 | 0.02229 | 3.165041681 | 0.046627448 |
| f__Streptococcaceae.g__Lactococcus.s__Lactococcus_sp__LG606 | 0.000006659 | 8.853E-07 | 2.19031769 | 0.043261211 |
| f__Streptococcaceae.g__Streptococcus.s__Streptococcus_gallolyticus | 0.02982 | 0.009019 | 2.132245121 | 0.01250128 |

Supplementary Table 4. The performances of all prediction models.

Random Forest Analysis

Patients with UDM vs HC

Model 1: microbiome (5 species)

Model 2: microbiome (5 species) + serum metabolome (7 serum metabolites)

Model 3: microbiome (5 species) + serum metabolome (7 serum metabolites) +clinical parameters (3)

| Features | | | | |
| --- | --- | --- | --- | --- |
| Model 1 | Model 2 | | Model 3 | |
| s__Clostridium_sp._AF24-2LB | s__Clostridium_sp._AF24-2LB | 2-hydroxyisocaproate | s__Clostridium_sp._AF24-2LB | 2-hydroxyisocaproate |
| s__Clostridium_saudiense | s__Clostridium_saudiense | 3-hydroxyisovalerate | s__Clostridium_saudiense | 3-hydroxyisovalerate |
| s__Lachnospira_eligens | s__Lachnospira_eligens | D-Mannose | s__Lachnospira_eligens | D-Mannose |
| s__Clostridium_sp._AM32-2 | s__Clostridium_sp._AM32-2 | TG 18:0_18:0_18:1 | s__Clostridium_sp._AM32-2 | TG 18:0_18:0_18:1 |
| s__Granulicatella_sp. | s__Granulicatella_sp. | TG 17:0_18:1_18:1 | s__Granulicatella_sp. | TG 17:0_18:1_18:1 |
|  |  | TG 16:0_17:0_18:1 | HBA1C | TG 16:0_17:0_18:1 |
|  |  | TG 18:0_18:2_19:1 | FCP | TG 18:0_18:2_19:1 |
|  |  |  | UA |  |
| HC vs UDM: AUC = 0.66 | HC vs UDM: AUC = 0.73 | | HC vs UDM: AUC = 0.94 | |

HC, healthy control; UDM, unclassified diabetes; AUC: area under curve.
